# Get the unbalance right: asymmetric transfer effects in cognitive offloading

**DOI:** 10.1186/s41235-026-00722-0

**Published:** 2026-03-26

**Authors:** Irene Florean, Marta Stragà, Timo Mäntylä, Fabio Del Missier

**Affiliations:** 1 Department of Life Sciences, University of Trieste, Via Weiss 2, 34128 Trieste, TS Italy; 2 Department of Psychology, Stockholm University, Albanovägen 12, 106 91 Stockholm, Sweden

**Keywords:** Cognitive offloading, Planning, Route planning, Transfer, Cognitive strategies, Working memory

## Abstract

Research has shown that cognitive offloading improves immediate performance in perception, short-term memory, and prospective memory, but also has negative side effects, such as poorer memory for offloaded information. However, the transfer-related consequences of cognitive offloading on subsequent performance are still largely unknown. In two experiments, we investigated these consequences in a planning task in which participants were asked to find the shortest route connecting locations on a map, given some constraints on the order of visit. Transfer designs were used to assess the effects of training with or without the opportunity to offload cognition on subsequent planning performance when this opportunity was removed or offered. In Experiment 1, participants were either allowed to use the pen while planning on the map and spontaneously devise offloading strategies or they were prevented from doing so. In Experiment 2, visual aids supporting the offloading strategies observed in Experiment 1 were embedded into the maps in some conditions and not in others. The results showed that the negative effect of offloading removal exceeded the positive effect of its introduction, supporting the hypothesis that the transition from a simpler and more intuitive visual planning strategy to a more demanding incremental planning strategy entails higher strategy switching costs and challenges reconfiguration processes more than the reverse transition. More generally, the results show that the opportunity to offload cognition in complex tasks can affect the strategies used by participants and this can have cognitive consequences for the subsequent adoption of different strategies when environmental conditions change.

**Supplementary Information:**

The online version contains supplementary material available at 10.1186/s41235-026-00722-0.

## Introduction

Cognitive offloading has been defined as “the use of physical actions to alter the information processing requirements of a task so as to reduce cognitive demand” (Risko & Gilbert, 2016, p. 676). Offloading can reduce cognitive load through the execution of physical actions with no changes to the physical world (e.g., counting on the fingers, tilting the head to read a rotated text, e.g., Carlson et al., 2007; Risko et al., 2014) or through actions affecting the state of objects or environments (e.g., setting a reminder or writing a note; Eskritt & Ma, 2014; Gilbert et al., 2023). A particularly relevant aspect of offloading on external artifacts is represented by its short- and long-term consequences.

Although offloading generally aids immediate task performance and reduces cognitive effort (Florean et al., 2025a; Gilbert et al., 2023; Gilbert, 2015a), it can have negative side effects, like poorer memory of externally saved statements after typing them (Sparrow et al., 2011, but see Schooler & Storm, 2021 for boundary conditions) or poorer memory of objects after picture taking (Henkel, 2014). While the offloading literature focused on the incidental side effects of offloading after performing a task, little attention has been paid to what happens when a task is initially performed with offloading and afterward without offloading (or vice versa). Incidental side effects and transfer effects are different, and both are theoretically and practically relevant (e.g., Kosmyna et al., 2025; Sachdeva & Gilbert, 2020; Scarampi & Gilbert, 2020; Sparrow et al., 2011). Moreover, cognitive offloading and its consequences have been mainly studied in perception, short-term memory, intention offloading, and episodic memory (Gilbert, 2024; Gilbert et al., 2023; Risko & Gilbert, 2016;), while other important cognitive domains, such as planning, have been almost completely neglected (see Florean et al., 2025a).

In this article, we report the results of two experiments conducted to investigate the transfer-related consequences of cognitive offloading in a route planning task, thus filling a significant gap in the literature. The experiments assessed both the consequences of removing the opportunity to offload cognition after a stage in which this opportunity was offered and the consequences of providing the opportunity to offload cognition after a stage in which this opportunity was not offered. To the best of our knowledge, this is the first study with these aims within the cognitive offloading literature, which has scantly investigated the consequences of offloading with transfer designs (Scarampi & Gilbert, 2020) and never did this in a planning domain.

Beyond extending the investigation of the cognitive consequences of offloading to a new set of transfer-related situations and to a new domain, these experiments can offer novel general insight into strategic changes in cognitive planning associated with variations between the learning conditions and the test conditions and their effects on performance (Lemaire & Lecacheur, 2010; O'Hara & Payne, 1998; Schillemans et al., 2010).

In the next two sections, we present a focused review of research on cognitive offloading, including studies most relevant to our aims and hypotheses. We then introduce our two experiments and the rationale underlying the hypotheses tested. Next, we describe the experiments and their results. We conclude with a discussion highlighting the theoretical and practical implications of our findings, as well as the limitations of the research and potential future directions.

### Immediate and short-term consequences of cognitive offloading

Gilbert et al. (2023) summarized results from studies on intention offloading, which involves setting external reminders or cues in advance to trigger delayed intentions at the appropriate time (e.g., writing a sticky note and placing it in plain sight to remember to do something). They observed that intention offloading is used more often when the task to be performed is more cognitively demanding. Offloading usually improves immediate performance, but individuals tend to offload more than necessary (e.g., Fröscher et al., 2022) and to offload more if this was previously allowed vs. not allowed, exposing a perseveration effect (see also Scarampi & Gilbert, 2020). Gilbert et al. (2023) identified as potential predictors of the tendency to offload cognition participants’ lower cognitive abilities and lower confidence in their performance (see also Gilbert, 2015b; Gilbert et al., 2020), thus highlighting the role of individual differences. Offloading has been studied also in a limited number of visuospatial tasks. These studies observed improved performance when participants can actively change the spatial arrangement of elements of a complex graphical representation to extract information (Moritz et al., 2018), and when children and adults can rotate external elements to solve problems that require mental rotation (Armitage et al., 2020; Kirsh & Maglio, 1994).

Research also highlighted negative side effects of offloading, such as poorer memory of externally stored verbal and visual contents (e.g., Henkel, 2014; Sparrow et al., 2011) and overconfidence in one’s own knowledge after the use of search engines (e.g., Fisher et al., 2015; Ward, 2021). However, these findings have not always been replicated (see e.g., Camerer et al., 2018; Hesselmann, 2020). The worrisome risk of long-term weakening of unpracticed abilities has been pointed out (e.g., Carr, 2008; Dahmani & Bohbot, 2020), but concerns about long-term negative outcomes are currently mainly speculative and more investigation is needed (see also Benge & Scullin, 2025; Kosmyna et al., 2025).

### External support and cognitive offloading in route planning

The use of GPS and navigation technologies has been considered as a strong form of visuospatial offloading (Risko & Gilbert, 2016), automating fundamental components of the wayfinding process and thus helping performance. However, some evidence highlighted also negative side effects of these technologies (e.g., Fenech et al., 2010; Gardony et al., 2013; for a review, see Brügger et al., 2019), with the limited attention paid to the surroundings leading to poorer spatial memory and wayfinding when the technology is unavailable (Gardony et al., 2015). According to Dahmani and Bohbot (2020), the intensive use of GPS technology may even impair spatial memory in the long term, but navigation technologies that promote learning of a cognitive map seem able to improve navigation while preserving spatial knowledge and memory (Liu et al., 2022, see also Brügger et al., 2019).

Beyond examining positive and negative effects, a few previous studies provided insight into how external support and cognitive offloading could affect route planning strategies. Wiener et al. (2009) asked participants to navigate the shortest path between a subset of several boxes placed on the floor in a room in a 3D variant of the traveling salesperson problem.^1^ For the first group of participants, the target boxes to be included in the solution were visually highlighted with a black marker, while for the second group they were indicated in a list. Wiener et al. observed shorter routes in the first group when the number of target boxes was larger. They explained this difference by referring to the application of an externally supported global planning strategy in the first group, with marked target locations directly accessible to perception. Conversely, when target locations were not visually marked, they had to be found and their positions maintained in working memory for planning. Thus, participants in the second group had to resort to a simplified but less effective incremental planning heuristic when the number of targets exceeded their working memory capacity. These findings agree with other studies on 2D maps, which pointed out that multiple-errands or path planning tasks can be solved with different strategies in different conditions (e.g., Fum & Del Missier, 2000a; Hayes-Roth, 1979), varying significantly in the demands they pose on cognitive planning vs. visual routines (Hayes-Roth & Thorndyke, 1979).

Following up this work, Florean et al. (2025a) carried out the first study on the spontaneous use of offloading while planning the shortest route to connect locations on 2D maps while satisfying ordering constraints. They manipulated map difficulty and the possibility for participants to offload cognition by allowing or not allowing them to use a pen on the map during planning. The study identified four offloading strategies spontaneously used by participants: (1) marking out the starting and arrival locations on the map with the pen; (2) marking out the locations to be visited; (3) marking out the visiting order constraints; (4) tracking the path planned on the map. Participants used more types of offloading strategies in the high (vs. low) difficulty maps and showed a better planning performance in the offloading (vs. no offloading) condition in the high-difficulty maps only. However, even in the low difficulty maps, cognition was offloaded, especially when participants solved the high-difficulty maps first, highlighting a perseveration effect. In the high-difficulty maps, the use of offloading strategies and offloading-supported planning performance were related with individual differences in visual search ability, while planning performance in the no offloading condition was related with individual differences in spatial working memory. Together with other findings, this indicated that participants in the different experimental conditions adopted different strategies, which require varying combinations of cognitive skills depending on whether opportunities to offload cognition are available or not.

This interpretation agrees with Wiener et al. (2009) and with studies in route planning in which the use of different kinds of strategies has been reported (Hayes-Roth, 1979; Hayes-Roth & Thorndyke, 1979). Indeed, participants in the offloading condition in Florean et al. (2025a) transformed the route planning task into a visual task similar to a traveling salesperson problem by marking out locations and constraints on the map with the pen, a task that can be solved very effectively by using visual strategies and routines (Burns et al., 2006; MacGregor et al., 2011; MacGregor & Omerod, 1996). Conversely, participants in the no offloading condition, who were not allowed to use the pen during planning, had to resort to an incremental planning strategy (Fum & Del Missier, 2000b; Hayes-Roth & Hayes-Roth, 1979; Reitter & Lebiere, 2010), mentally planning partial paths and memorizing the labels of the corresponding locations to report them later (Wiener et al., 2009).

Previous studies on convex hull models of the traveling salesperson problem suggest that the visual strategy fostered by cognitive offloading can be based on the perception of an approximate global route roughly connecting all the locations to be visited from the starting point to the arrival point, which can be readily and rapidly available via low-level global perceptual processes (MacGregor & Ormerod, 1996; MacGregor et al., 1999, 2000, see also Vickers et al., 2001). Once a sketch of this approximate solution is visually identified, locations to be visited can be connected sequentially from the starting point to the arrival point and the solution refined. Importantly, this strategy can be applied only when the locations to be visited are immediately apparent, such as when they are visually highlighted and there is no need to keep their positions in memory to distinguish them from the other locations on the map (Wiener et al., 2009). On the contrary, when locations to be visited do not visually stand out from distractors, an incremental and more demanding strategy needs to be used. In this case, the participants need to identify the positions of the locations to be visited on the map and keep them in memory, together with ordering constraints. As this stresses working memory capacity (Wiener et al., 2009), participants have to simplify the task (Tenebrink & Wiener, 2009), and this can be done by identifying regions or clusters of points to be connected and then plan iteratively at the level of a single region or cluster, beginning with the one closer to the starting point and then moving progressively toward the arrival point (see also Best & Simon, 2005; Hirtle & Gärling, 1992; Kong & Schunn, 2007; Vickers et al., 2003). Within each cluster, points can be connected using the nearest neighbor heuristics or other simplifying methods. Working memory can help to keep in mind both regions or cluster positions and the positions of locations within each cluster while planning locally.

### Experiments and hypotheses

The studies reviewed in the previous sections indicate that cognitive offloading may offer benefit in terms of immediate performance but may also have negative consequences for learning and memory. They also highlighted that the opportunity of offloading cognition can change the strategies used in route planning tasks, which have different cognitive requirements. Starting from this research, we conducted two experiments to investigate the bidirectional nature of transfer effects in cognitive offloading. In particular, we examined how allowing/not allowing the opportunity to use offloading strategies affected subsequent performance when this opportunity was either removed or introduced. While earlier offloading research primarily focused on episodic or prospective memory with relatively simple tasks, our research employed a more complex route planning task.

In Experiment 1, participants were free to spontaneously devise their own offloading strategies while performing the route planning task. The opportunity to offload cognition was provided by allowing (or not allowing) participants to use the pen while planning the routes on the maps, following Florean et al. (2025a). In Experiment 2, the strategies that participants spontaneously devised in Experiment 1 and Florean et al. (2025a) were supported by embedding specific visual aids into the maps, which highlighted the position of starting and arrival points and locations to visit, provided explicit ordering constraints on the map, and enabled notetaking to track the progress of the plan. The visual aids clarified which offloading strategies participants could employ, removing the need to discover them independently. Beyond representing a conceptual replication of Experiment 1, Experiment 2 allowed us to test the effectiveness of visual aids for cognitive offloading in the route planning domain.

The hypotheses were the same for both experiments. In accordance with prior research indicating that cognitive offloading typically enhances immediate task performance (e.g., Florean et al., 2025a; Gilbert et al., 2023; Gilbert, 2015a), we expected that removing the opportunity to offload cognition would impair route planning (H1) and that introducing such opportunity would improve it (H2). Crucially, we further expected that the decline in performance following the removal of offloading would be greater than the improvement following its introduction (H3).

H3 was grounded in evidence that cognitive offloading in the route planning task can qualitatively change the strategies employed to find the solution (see Section "External Support and Cognitive Offloading in Route Planning"). Therefore, we expected strategy shifts to occur after the introduction or removal of the opportunity to offload cognition, and that these shifts would produce asymmetric transfer effects. Specifically, we expected that switching from a spontaneous and primarily visual strategy to a more taxing working memory-based strategy after offloading removal would be more challenging than switching in the reverse direction after offloading introduction. Indeed, picking up the intuitive visual strategy should be easy when offloading cognition into the map transforms the planning task into a sort of traveling salesperson problem (Florean et al., 2025a), with limited interference from the demanding incremental planning strategy previously experienced. Conversely, having used such an effective and effortless visual strategy in the past can get more into the way when it cannot be used anymore, and the participants need to switch to a more thoughtful and demanding incremental planning strategy. In this case, the reconfiguration processes are expected to be more taxing (Lemaire & Lecacheur, 2010), also due to participants trying to apply or adapt the simpler intuitive strategy previously applied. Building on this line of thought, we also expected that, when participants had to switch from the offloading to the no offloading condition, individuals with better working memory capacity would better adapt and would embrace more easily the incremental planning strategy, thus exposing a less pronounced asymmetric transfer effect (H4).

In addition to the effects on performance, we further hypothesized that changing the availability of cognitive offloading would also change participants' perceptions of the task. In agreement with a previous study showing that participants who were given the opportunity to offload cognition perceived the task as less difficult and less effortful than those who were not given such opportunity (Florean et al., 2025a), we expected that removing the opportunity to offload cognition would lead to an increase in perceived task difficulty and effort (H5), whereas introducing this opportunity would lead to a decrease in these perceptions (H6). Considering that participants’ judgments of difficulty and effort are not always perfectly aligned with performance (Florean et al., 2025a), we did not have specific hypotheses about potential asymmetries in these judgments. An additional specific hypothesis was tested in Experiment 2, in which we expected no performance differences when all four offloading strategies or only two of them were supported by visual aids in the maps (H7). Considering that this hypothesis was formulated a priori, but after the consideration of the results of Experiment 1, we will introduce it together with Experiment 2.

## Experiment 1

In Experiment 1, participants were first presented with a training block of the route planning task (Florean et al., 2025a) and required to complete it under different between-participants conditions. A subset of participants was trained with the opportunity to offload cognition by freely using a pen on the map, whereas other participants were trained without such opportunity or asked to keep their hands still while planning. The latter condition prevented participants from offloading cognition onto the body by using their hands when external offloading via pen use was precluded (Risko & Gilbert, 2016). After the training block, participants completed two counterbalanced test blocks. In one block, participants performed the route planning task under the same conditions as in their training. In the other test block, participants who were trained with pen use were prevented from using it or asked to keep their hands still, while participants who were trained without the pen were allowed to use it.

### Method

#### Participants

An a priori power analysis indicated a sample size of 136 participants for our experimental 4 × 2 mixed design (see Section "Experimental Design") to detect a medium effect size for the between-factor effect (*f* = 0.25, α = 0.05, 1 − β = 0.80, *r*_repeated_measures_ = .50), as the one observed for the accuracy measure in high-difficulty maps in Florean et al. (2025a).^2^ We prudentially recruited 142 undergraduates for this study, in case any extreme cases or noncompliant participants needed to be excluded. Participants who did not adhere to the route planning task instructions (*n* = 4), and those who had missing data on measures from all the tasks included in the experiment (*n* = 1) were excluded from the analyses. The final sample consisted of 137 participants (84.67% female, age: *M* = 22.36, *SD* = 5.78). The study was approved by the Ethical Committee of the University of Trieste. Informed consent was obtained from each participant at the beginning of the experimental session and participants received course credits for their participation, regardless of their level of performance.

#### Experimental design

**Fig. 1:**
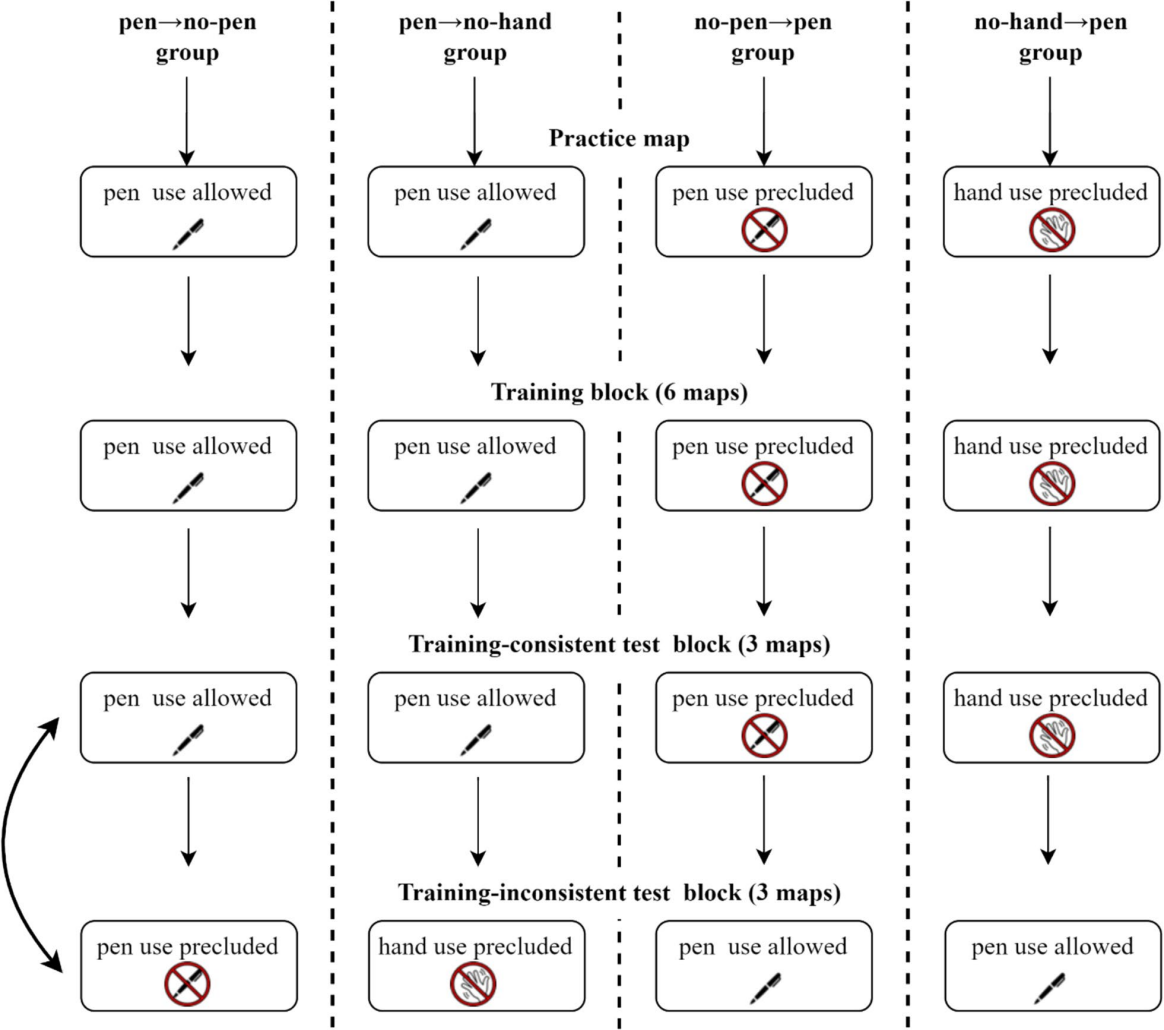
Overview of the design and main steps of the procedure in experiment 1. *Note.* Bidirectional arrows indicate the counterbalanced presentation of test blocks. For clarity, the figure does not show steps related to instructions, performance prediction and postdiction, and evaluation of perceived difficulty and effort (see Section “Procedure”)

The experiment followed a 4 (group: pen → no-pen *vs*. pen → no-hand *vs*. no-pen → pen *vs*. no-hand → pen) × 2 (test block: training-consistent *vs*. training-inconsistent) mixed design (see Fig. 1). All participants carried out a training block followed by two test blocks of the route planning task. The training condition was manipulated between participants, and one of the test blocks was performed under a condition different from the training. During the training block, two groups of participants were allowed to freely use a pen on the map sheets during the task (pen → no-pen and pen → no-hand groups). Another group was instructed to use the pen only to indicate the final routes (no-pen → pen group). A fourth group was additionally instructed to keep the hands still on the table until the final routes were fully identified and ready to be reported (no-hand → pen group).

After completing the training block, participants carried out two counterbalanced test blocks. In one test block, participants performed the task under the same condition as in the training block (training-consistent block). In the other block (training-inconsistent block), both groups trained with the pen were restricted from using it, with one of these groups also being restricted from using their hands. Conversely, the groups initially not allowed to use the pen, or both the pen and the hands, were now allowed to use the pen.

#### Procedure

At the beginning of the experimental sessions, participants read and accepted the informed consent form and were then randomly assigned to one of four groups: pen → no-pen (*n* = 35), pen → no-hand (*n* = 36), no-pen → pen (*n* = 33), no-hand → pen (*n* = 33). Participants read the instructions for the training block of the route planning task, completed a practice map, and estimated how much they thought the routes they were about to plan would deviate from the shortest possible routes in the upcoming block.^3^ The instructions for the training block were identical across all groups, with the following exceptions. Participants in the pen → no-pen group and the pen → no-hand group were informed that they could use the pen on the map sheet at any point during the task, even before recording their final route. Participants in the no-pen → pen group were instructed to use the pen exclusively to record the final route in the designated response area, with no instructions regarding hand use. Participants in the no-hand → pen group were also instructed to use the pen exclusively for recording the final route in the designated response area but were additionally required to keep their hands still on two blue plastic rectangles attached to the table on the two sides of the booklet until they had fully planned their route and were ready to record it. After completing the maps for the training block, participants reported how much they believed they had deviated from the shortest routes, as well as how difficult and effortful they found the completion of the training block. Participants were then given a brief filler personality questionnaire (Woo et al., 2014) to introduce a short delay between the training and the test blocks, which took a median of 42.53 s to complete.

Once they completed the questionnaire, participants proceeded to the test blocks, which were presented in a counterbalanced order within each group. The instructions for the training-consistent block informed participants that they had to perform the route planning task exactly as in the training stage. The instructions for the training-inconsistent block informed participants that they had to perform the route planning task under a condition different from the one practiced during the training, with specific differences explained according to the condition. Both test blocks were delivered following a procedure like the one of the training block, consisting of instructions, performance prediction, route planning task, retrospective judgment of performance, and evaluation of perceived difficulty and effort. Finally, participants completed a computerized visual search task (Multiple Features Target Cancellation task, MFTC; Marra et al., 2013), answered demographic questions, and performed a spatial working memory task (Symmetry Span Task, Kane et al., 2004). The total duration of the procedure was approximately 70–80 min (see Fig. 1 for a graphical summary). A debriefing session was held after data collection, with disclosure of the full aims of the study and the provision of further information.

#### Tasks and measures

We used the paper-and-pencil route planning task from Florean et al. (2025a), which presents a series of maps of abstract locations, requiring participants to find the shortest possible path from a given starting place to a given arrival place, visiting specific intermediate locations, and following prescribed ordering rules. This task employs artificial maps to minimize potential confounds due to familiarity with real places (Muffato & Meneghetti, 2021) or peculiar features of the stimuli (e.g., known landmarks such as church, city hall, etc.). Locations on the maps are labeled with single letters for the same reasons and to control for potential semantic/lexical confounds (see, e.g., Wiener & Mallot, 2003).

**Fig. 2:**
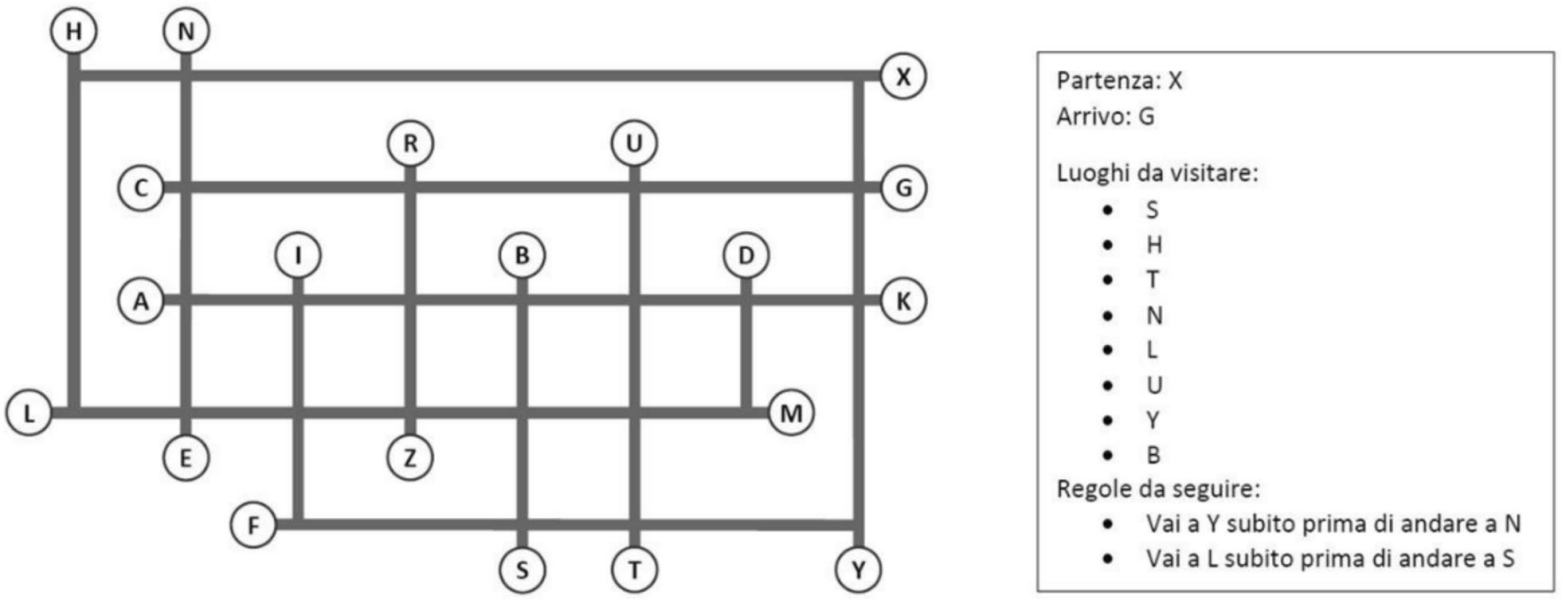
Example map of the route planning task. *Note.* The translation of the right panel is as follows*: Partenza: X* is the starting place (X), *Arrivo: G* is the arrival place (G), *Luoghi da visitare: S, H, T, N, L, U, Y, B* are the locations to be visited (S, H, T, N, L, U, Y, B), and *Regole da seguire: Vai a Y subito prima di andare a N, Vai a L subito prima di andare a S* are the two ordering rules stating that location Y must be visited immediately before location N and location L must be visited immediately before location S

In particular, we employed 12 high-difficulty maps used in Florean et al. (2025a) to create two blocks of six maps comparable in terms of speed and accuracy to be assigned to the training and test blocks (for an example, see Fig. 2**)**. Each map presented a starting place, an arrival place, eight intermediate locations to be visited, two ordering rules (each constraining the order of visit of a pair of intermediate locations), and ten distractor locations. Participants were provided with a booklet containing a series of layered sheets, each displaying a map along with its specific instructions in the rightmost area (e.g., starting and arrival places, locations to be visited, and ordering rules to follow). Participants were instructed to plan the shortest route as quickly and accurately as possible and, only after fully identifying their solution, to report it in a designated response frame located beneath the map. This required them to write down the locations (letters) in the exact order as they planned to visit them. Once the entire route was reported, participants were required to flip the sheet and place it in a box on their right before moving on to the next map, and they were not allowed to return to previously completed maps.

Following Florean et al. (2025a), the shortest path for each map among the ones completed without errors by all participants was identified (i.e., including the starting and arrival places and the locations to be visited, and respecting the ordering rules). Planning accuracy for each map solved without errors was measured as the proportional deviation above the shortest route, namely the proportion by which the planned route length exceeded the shortest route for that map (see, e.g., MacGregor & Ormerod, 1996), computed as follows: *proportional deviation* = (actual route length—shortest route length) / shortest route length. A higher value of this measure indicates a greater deviation from the shortest route and a zero value indicates no deviation. We used a relative evaluation of performance because, to date and to the best of our knowledge, no automated algorithm is available for computing the shortest route for visiting a series of places when the order of visits of some of them is constrained. To measure the completion time of each map solved without errors, the time interval between the flip of the previous map sheet and the flip of the given map sheet was recorded.

All maps completed under the condition allowing pen use were evaluated by an expert rater who classified the offloading strategies employed by participants using the taxonomy from Florean et al. (2025a), which proved to be highly reliable.^4^ This taxonomy identifies four possible categories of offloading strategies: (1) marking starting and arrival locations; (2) marking intermediate locations to be visited; (3) marking ordering constraints; and (4) keeping track of the plan. As in Florean et al. (2025a), no additional offloading strategies were identified in the present study. Maps were individually assessed for the presence or absence of each offloading strategy. Each map was also evaluated for the total number of offloading strategies implemented, with scores ranging from 0 (no strategies used) to 4 (all strategies used). The number of strategies employed when pen use was allowed was then averaged across maps belonging to the same block (i.e., training block, training-consistent test block, and training-inconsistent test block). After finishing each block of maps, participants were also asked to rate the difficulty of the maps they had just completed and the amount of effort they had put into completing them, using a 7-point scale ranging from *very little* to *very much*.

To assess spatial working memory capacity, we employed the Symmetry Span task (Kane et al., 2004) from the Attention & Working Memory Lab (https://englelab.gatech.edu/). In each trial, participants viewed a series of 8 × 8 matrices with black-and-white squares and determined whether each matrix was symmetrical along its vertical axis (process component). Following each symmetry judgment, a red square was briefly displayed within a 4 × 4 matrix (storage component). Each sequence comprised 2 to 5 matrices and concluded with a 4 × 4 matrix where participants were required to click on the squares corresponding to the previously shown red squares according to their order of appearance. Before the test trials, participants completed separate practice sessions for the process component, the storage component, and the full task. During the test trials, if the time spent on a symmetry judgment exceeded the average practice time by more than 2.5 standard deviations, the program automatically presented the subsequent red square and recorded the symmetry judgment as an error. Performance was assessed using the partial score, which considers all correctly recalled red squares (Oswald et al., 2015; Redick et al., 2012).^5^ Data were analyzed using Jamovi (version 2.6.44).

### Results

#### Preliminary analyses and manipulation check

For each performance variable of the route planning task, data was trimmed within the groups of participants and blocks of maps. Values that were 3 *SD*s above or below the mean were replaced by the mean ± 3 *SD*s (for a similar procedure, see Del Missier et al., 2021; Florean et al., 2025a; Miyake et al., 2000). As previously specified, one participant was excluded from the subsequent analyses due to missing data on measures from all the tasks included in the experiment. In the case of the MFTC and Symmetry Span tasks, data trimming was conducted on the entire sample. Table 1 presents the descriptive statistics for the performance measures of the route planning task by groups and blocks.^6^ Groups did not differ significantly on gender, χ^2^(3) = 5.40, *p* = .144, visual search skills, *F*(3,133) = 2.38, *p* = .072, and spatial working memory, *F*(3,132) = 1.38, *p* = .25.

**Table 1:** Descriptive statistics of route planning measures in experiment 1

Block	Map completion time			Proportional deviation above the shortest route			Perceived Task Difficulty			Perceived Task Effort		
	Training	Consistent Test	Inconsistent Test	Training	Consistent Test	Inconsistent Test	Training	Consistent Test	Inconsistent Test	Training	Consistent Test	Inconsistent Test
	M (SD)	M (SD)	M (SD)	M (SD)	M (SD)	M (SD)	M (SD)	M (SD)	M (SD)	M (SD)	M (SD)	M (SD)
*Group*												
Pen → no-pen	188.17	162.34	237.68	0.12	0.09	0.16	4.40	3.74	5.57	4.46	4.06	5.46
	(67.31)	(51.12)	(66.70)	(0.06)	(0.08)	(0.13)	(1.12)	(1.24)	(1.40)	(1.24)	(1.43)	(1.38)
Pen → no-hand	197.59	164.49	231.42	0.10	0.11	0.18	4.25	4.11	5.61	4.33	4.39	5.78
	(55.35)	(51.78)	(85.73)	(0.05)	(0.09)	(0.14)	(1.13)	(1.17)	(0.99)	(1.24)	(1.36)	(1.02)
No-pen → pen	232.20	209.13	179.01	0.12	0.14	0.12	5.18	4.88	4.06	5.45	5.03	4.00
	(65.34)	(53.34)	(42.65)	(0.07)	(0.12)	(0.09)	(0.95)	(1.24)	(1.14)	(1.00)	(1.29)	(1.25)
No-hand → pen	203.41	204.13	173.94	0.12	0.16	0.12	5.09	5.09	3.30	5.33	5.12	3.40
	(65.43)	(77.51)	(51.98)	(0.09)	(0.14)	(0.11)	(0.98)	(0.88)	(1.19)	(0.78)	(1.08)	(1.25)

Completion times are reported in seconds

As a check for our offloading manipulation, we assessed whether participants actually used offloading strategies when allowed to use the pen in the test blocks. To this end, we conducted a series of one-sample t-tests for each group to test whether the mean number of strategies employed by participants in the test blocks with pen use allowed was significantly different from zero. The hypothesis was corroborated in each group. Participants in the pen → no-pen group employed an average of 3.52 strategies (*SD* = 0.73), significantly above zero, *t*(34) = 28.60, *p* < .001, *d* = 4.83. Similarly, those in the pen → no-hand group employed an average of 3.35 strategies (*SD* = 0.94), *t*(35) = 21.49, *p* < .001, *d* = 3.58. Participants who were allowed to use the pen for the first time during the test stage also made significant use of offloading strategies: the no-pen → pen group employed an average of 2.46 strategies (*SD* = 0.87), *t*(32) = 16.20, *p* < .001, *d* = 2.82, while the no-hand → pen group employed 2.56 strategies on average (*SD* = 1.22), *t*(32) = 12.02, *p* < .001, *d* = 2.09. Therefore, participants in all conditions employed offloading strategies when pen use was allowed during planning (for additional analyses, see Supplementary Materials, section S3).

Given that the primary aim of Experiment 1 was to examine how training in the route planning task under different conditions affects subsequent performance in the two test blocks where the initial condition was either maintained or changed, the following analyses will focus on test blocks. Analyses for the training block are presented in Supplementary Materials (section S4).

#### Transfer effects on route planning task performance

To test our hypotheses related to transfer effects, we conducted a series of 4 (pen → no-pen *vs*. pen → no-hand *vs*. no-pen → pen *vs*. no-hand → pen groups) × 2 (training-consistent *vs.* training-inconsistent test blocks) mixed ANOVAs on the performance measures of the maps completed without errors, followed by the four key planned comparisons across test blocks.^7^ The results reported in the following sections did not change after controlling for test block order (see Supplementary Materials, section S5).

##### Proportional deviation above the shortest route

**Fig. 3:**
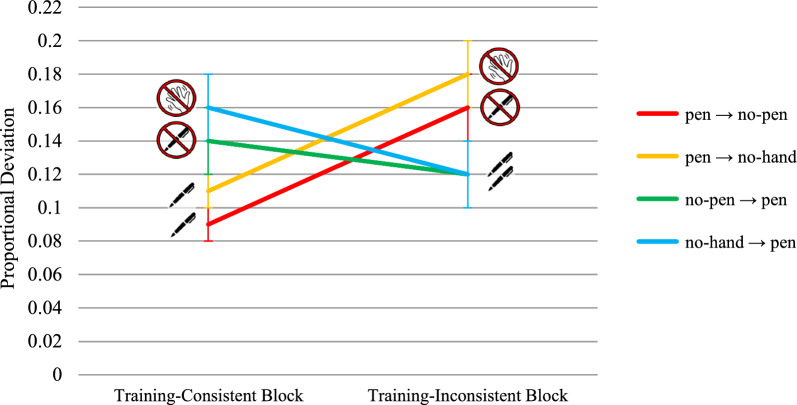
Transfer Effects on Proportional Deviation above the Shortest Route in Experiment 1. *Note*. Bars represent standard errors. Higher scores indicate worse performance

A significant group × test block interaction was found on the proportional deviation above the shortest route, *F*(3,132) = 8.42, *p* < .001, η^2^_p_ = 0.16, while neither the main effect of group nor the main effect of block reached statistical significance, *F*s < 1.88, *p*s > .173 (see Fig. 3 and Table 1 for descriptive statistics). Both the pen → no-pen group and the pen → no-hand group planned significantly longer routes in the training-inconsistent block than in the training-consistent block, *t*(132) = 2.99, *p* = .003, *d* = .52, and *t*(132) = 3.43, *p* < .001, *d* = .50, respectively. In contrast, only the no-hand → pen group planned significantly shorter routes in the training-inconsistent block compared to the training-consistent block,* t*(132) = -2.31, *p* = .022, *d* = -.39, whereas the no-pen → pen group showed a non-significant improvement, *t*(132) = -1.22, *p* = .225, *d* = -.26. Therefore, participants who were previously trained with pen use allowed were significantly and negatively affected by the restriction of pen use during the subsequent testing stage, while among participants prevented from using the pen in the training stage, only those who were additionally precluded from using their hands showed a significant but small improvement.

These results were only in partial agreement with our first two hypotheses, as removing the opportunity to offload cognition impaired route planning accuracy (H1) but introducing such an opportunity did not always lead to a significant improvement (H2). However, the fact that removing pen use resulted in longer planned routes, whereas introducing it did not consistently result in shorter routes, provides support for the asymmetric transfer effect hypothesis (H3). Nonetheless, since the hypothesis on moderation by working memory capacity (H4) builds upon all the previous ones, it could not be tested on this variable.

##### Map completion time

**Fig. 4:**
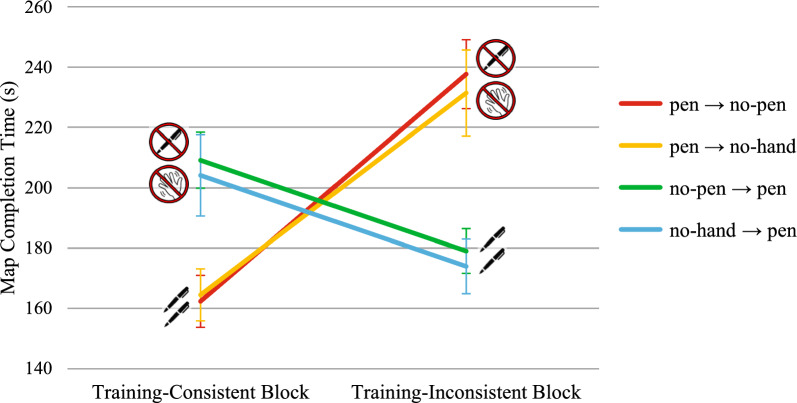
Transfer Effects on Map Completion Time in Experiment 1. *Note*. Bars represent standard errors. Higher scores indicate worse performance. Time is expressed in seconds

The mean time required to complete a map was significantly affected by the test block, *F*(1,132) = 16.80, *p* < .001, η^2^_p_ = 0.11, while the main effect of group was not statistically significant, *F*(3,132) = 0.28, *p* = .843, η^2^_p_ = 0.01. The group × test block interaction was also statistically significant, *F*(3,132) = 34.66, *p* < .001, η^2^_p_ = 0.44 (see Fig. 4 and Table 1 for descriptive statistics). Participants in both the pen → no-pen group and the pen → no-hand group needed more time to complete maps in the training-inconsistent block compared to the training-consistent block, *t*(132) = 7.53, *p* < .001, *d* = 1.40, and *t*(132) = 6.96, *p* < .001, *d* = 1.03, respectively. Additionally, both participants in the no-pen → pen group and the no-hand → pen group spent significantly less time completing maps in the training-inconsistent block than in the training-consistent block, *t*(132) = -3.00, *p* = .003, *d* = .65, and *t*(132) = -3.01, *p* = .003, *d* = .48, respectively. Therefore, removing the opportunity to offload cognition significantly increased map completion time, in agreement with H1, and introducing such opportunity decreased it, in agreement with H2.

**Fig. 5:**
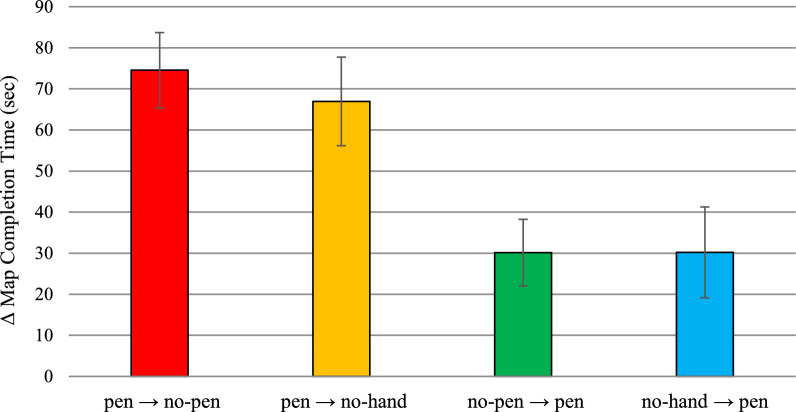
Extent of Slowdown/Speedup in Absolute Value in Map Completion Time in the Test Block after Removal/Introduction of Pen Use as a Function of the Group in Experiment 1. *Note*. Bars represent standard errors

According to the asymmetric transfer hypothesis (H3), when comparing the training-inconsistent block to the training-consistent block, the degree of slowdown showed by groups trained with pen use should exceed the degree of speedup exhibited by groups trained without pen use. To test this hypothesis, we first calculated for each group the difference in average map completion times (Δ map completion times) between the test block that precluded pen use and the test block that allowed pen use for each group. A higher Δ map completion time indicates a greater slowdown after pen removal for groups trained with pen use and a greater speedup after pen introduction for groups trained without pen use. A one-way ANOVA on Δ map completion time revealed significant between-group differences, *F*(3,132) = 5.68, *p* = .001, η^2^_p_ = 0.11 (see Fig. 5). Participants in the pen → no-pen group (*M* = 74.54 s, *SD* = 53.35 s) had significantly higher Δ map completion times than those in both the no-pen → pen group (*M* = 30.12 s, *SD* = 46.64 s), *t*(132) = 3.15, *p* = .002, *d* = .77, and the no-hand → pen group (*M* = 30.19 s, *SD* = 63.52 s), *t*(132) = 3.15, *p* = .002, *d* = .77. Similarly, participants in the pen → no-hand group (*M* = 66.93 s, *SD* = 64.73 s) had significantly higher Δ map completion times than those in the no-pen → pen group, *t*(132) = 2.65, *p* = .009 *d* = .64, and the no-hand → pen group, *t*(132) = 2.64, *p* = .009 *d* = .64. The remaining between-group comparisons were not statistically significant (*t*s <|0.56|, *p*s > .581).

**Fig. 6:**
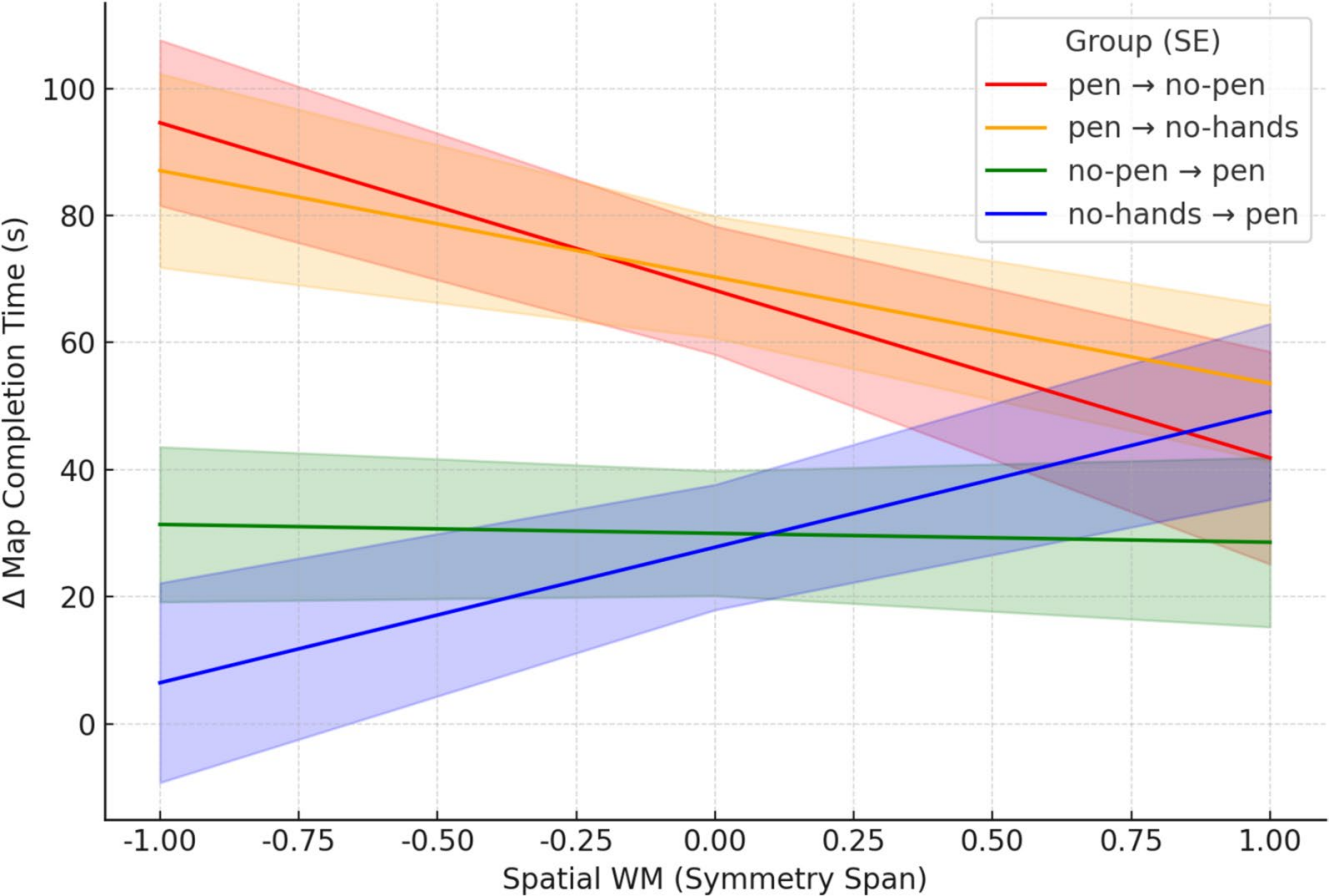
Extent of Slowdown/Speedup in Absolute Value in Map Completion Time in the Test Block after Removal/Introduction of Pen Use as a Function of Spatial Working Memory and Group in Experiment 1. *Note*. The values on the x axis represent Symmetry Span scores standardized as Z-scores

We expected that, when participants had to switch from the offloading to the no offloading condition, individuals with better working memory capacity would better adapt to the removal of the opportunity to offload cognition (H4). To test this hypothesis, we conducted a moderation analysis using General Linear Models (GLMs) on Δ map completion times between test blocks allowing and precluding pen use. The GLM included as predictors of Δ map completion times the group, the spatial working memory score (Symmetry Span), and their interaction. The interaction between group and spatial working memory was statistically significant, *F*(3, 127) = 3.65, *p* = .014, η^2^_p_ = .08. The group was a significant predictor, *F*(3,127) = 5.66, *p* = .001, η^2^_p_ = .12, but not the spatial working memory score, *F*(1,127) = 1.30, *p* = .256, η^2^_p_ = .01 (see Fig. 6).

Simple effects analyses showed that groups differed in Δ map completion time when spatial working memory scores were 1 *SD* below the mean, *F*(3,127) = 8.98, *p* < .001, η^2^_p_ = .18, and at the mean, *F*(3,127) = 5.66, *p* = .001, η^2^_p_ = .12, but not 1 *SD* above the mean, *F*(3, 127) = 0.70, *p* = .555, η^2^_p_ = .02. Specifically, when spatial working memory scores were 1 *SD* below the mean, participants in the pen → no-pen group had significantly greater Δ map completion times than those in the no-pen → pen group, *t*(127) = 3.54, *p* < .001, *d* = .87, and the no-hand → pen group, *t*(127) = 4.32, *p* < .001, *d* = 1.06, but did not differ significantly from those in the pen → no-hand group, *t*(127) = 0.38, p = .708, *d* = .09. At the spatial working memory mean, participants in the pen → no-pen group showed higher Δ map completion times than those in the no-pen → pen group, *t*(127) = 2.72, *p* = .007, *d* = .67, and the no-hand → pen group, *t*(127) = 2.87, *p* = .005, *d* = .71, but not from those in the pen → no-hand group, *t*(127) = -0.15, *p* = .881, *d* = .04. When spatial working memory scores were 1 SD above the mean, no significant between-group differences in Δ map completion times were observed (*t*s <|0.63|, *p*s > .535). The pattern of results did not change when using the pen → no-hand group as the reference group (see Supplementary Materials, section S6).

To summarize, in the transition to the training-inconsistent test block, participants trained with the pen experienced a slowdown greater than the speedup exhibited by participants trained without the pen (H3), except when they had higher spatial working memory, consistent with the hypothesis that working memory capacity moderates the asymmetric transfer effect (H4).

#### Transfer effects on perceived task difficulty and effort

We tested the hypothesis that the removal of pen use would lead to an increase in perceived task difficulty and effort (H5), while its introduction would result in a decrease in these perceptions (H6). We summarize the results of the analyses here; full details are reported in Supplementary Materials, section S7. Removing the option to offload cognition via pen use during the test stage increased perceived task difficulty (post-hoc comparisons: *t*s > 6.66, *p*s < .001) and effort (post-hoc comparisons: *t*s > 5.32, *p*s < .001) among participants trained with this option, consistent with H5. Conversely, introducing pen use led to a decrease in both perceived task difficulty (post-hoc comparisons: *t*s > 3.47, ps < .001) and effort (post-hoc comparisons: *t*s > 3.80, *p*s < .001) among participants trained without pen use, consistent with H6.

### Discussion

The results of Experiment 1 fully supported the hypothesis that removing the opportunity to offload cognition impairs route planning (H1) and partially supported the hypothesis that introducing such an opportunity improves route planning (H2). We observed the expected asymmetric transfer effects on deviation above the shortest route and map completion time (H3), with the latter being moderated by individual differences in spatial working memory (H4). Finally, removing the option to offload cognition led to an increase in perceived task difficulty and effort (H5), whereas introducing this option led to a decrease in these perceptions (H6).

The findings highlighted the differential cognitive consequences of switching between conditions, which we interpret as reflecting the greater difficulty of abandoning an established externalized strategy for an internal one. Notably, the moderation analysis revealed that participants trained with the pen were better able to cope with its removal when they had higher spatial working memory, suggesting that individual differences in cognitive abilities can buffer the negative effects of strategy shifts.

Task perception also shifted alongside performance. Participants trained with the pen perceived the task as more difficult and effortful when offloading was removed, while those trained without the pen found the task easier and less effortful once offloading became available. These changes in perception were consistent with our expectations, as removing the external support likely increased working memory load, making the task subjectively more challenging. In contrast, introducing the pen likely reduced cognitive load for participants previously deprived of offloading, leading to perceptions of lower difficulty and effort.

## Experiment 2

Experiment 2 aimed to extend the investigation of transfer effects on route planning by examining the impact of removing or introducing into the maps visual aids directly supporting the offloading strategies observed in Experiment 1 and in Florean et al. (2025a). According to the condition to which they were randomly assigned, participants were trained with visual aids for all four offloading strategies, with visual aids for two strategies only, or without visual aids. Then, they completed a test block under conditions that differed from their respective training, except for the control group, which completed both blocks without visual aids.

Our hypotheses were as in Experiment 1. We expected to observe negative effects following the removal of visual aids for offloading in the test block (H1), positive effects following their introduction (H2), and the asymmetric transfer effect of visual aids removal vs. introduction (H3) moderated by individual differences in working memory (H4). However, in Experiment 2 we focused on verbal working memory moderation, assessed by the backward digit span (Monaco et al., 2015), to examine an additional potential role of working memory in our task. Indeed, when no offloading aids are available, verbal working memory should be relevant for encoding and maintaining the solution route in chunks of letters during incremental planning, to report it later (see also Florean et al., 2025a). Based on our interpretation of the asymmetric transfer effects, we hypothesized that participants initially trained with visual aids would experience a less pronounced slowdown in solution-typing time after the removal of the aids if they had better verbal working memory. The test of this hypothesis in Experiment 2 was made possible by the clearer separation of the planning stage from the solution-typing stage in two different screens of the Qualtrics interface, unlike what happened in Experiment 1, in which participants used the same physical map sheet to plan and write the solution. We also expected that removing visual aids for offloading strategies would increase perceived task difficulty and effort (H5), while introducing them would lead to a decrease in these perceptions (H6).

An additional aim of Experiment 2 was to investigate whether providing visual aids for all four strategies or for two of them only would lead to differences in route planning performance. In Experiment 1, participants trained with the pen used more offloading strategies than those trained without it during the test block allowing pen use. Specifically, participants trained with the pen marked out more frequently ordering rules and kept an external track of their plan, whereas no significant differences were observed between groups in marking out starting and arrival locations or intermediate locations (see Supplementary Materials, section S3). However, this greater reliance on offloading strategies did not improve route planning performance, suggesting a "more than optimal" use of offloading strategies (e.g., Gilbert et al., 2020; see also Gilbert et al., 2023). Therefore, we hypothesized that participants performing the route planning task under the full offloading condition, in which all four strategies were externally supported, would not outperform those in the partial offloading condition, in which only the starting and arrival places and the intermediate locations were visually highlighted (H7).

### Method

#### Participants

An a priori power analysis, following Florean et al. (2025a) and Experiment 1, indicated that a minimum of 160 participants were required to detect a medium between-factor effect size (*f* = 0.25, α = 0.05, 1 − β = 0.85, *r*_repeated_measures_ = .40 from Experiment 1) in our 5 × 2 mixed experimental design (see Sect. "Experimental Design"). A total of 167 undergraduates were prudentially recruited in the study (85.63% female, age: *M* = 20.37, *SD* = 2.96), in case any extreme cases or noncompliant participants needed to be excluded.^8^ The study was approved by the Ethical Committee of the University of Trieste, and participants provided informed consent before completing the experimental session online. Course credits were granted for participation, regardless of performance level.

#### Experimental design

The experiment was carried out online. All participants completed an individual experimental session, consisting of a training block of the route planning task, followed by a test block. Participants were randomly assigned to one of five groups: one group trained with visual aids for all four the offloading strategies embedded in the maps (full offloading condition), another group trained with visual aids for two offloading strategies embedded in the maps (partial offloading condition), and three groups trained with maps without aids for offloading strategies (no offloading condition). After completing the training block, participants moved to the test block. For participants in the groups trained under the full and the partial offloading conditions, visual aids for offloading strategies were removed, requiring participants to complete the test block under the no offloading condition. Conversely, one group that was trained under the no offloading condition had all four visual aids available in the maps during the test block (shift to full offloading), and another group started from the no offloading condition and had maps with two visual aids available at the test stage (shift to partial offloading). The final group trained under the no offloading condition continued without any visual aid in the test block, serving as the control group. This setup resulted in a 5 (group) × 2 (block: training *vs*. test) mixed experimental design. Participants were randomly assigned to the five groups: full offloading → no offloading (*n* = 34); partial offloading → no offloading (*n* = 33); no offloading → full offloading (*n* = 34); no offloading → partial offloading (*n* = 34); no offloading → no offloading (*n* = 32).

#### Procedure

**Fig. 7:**
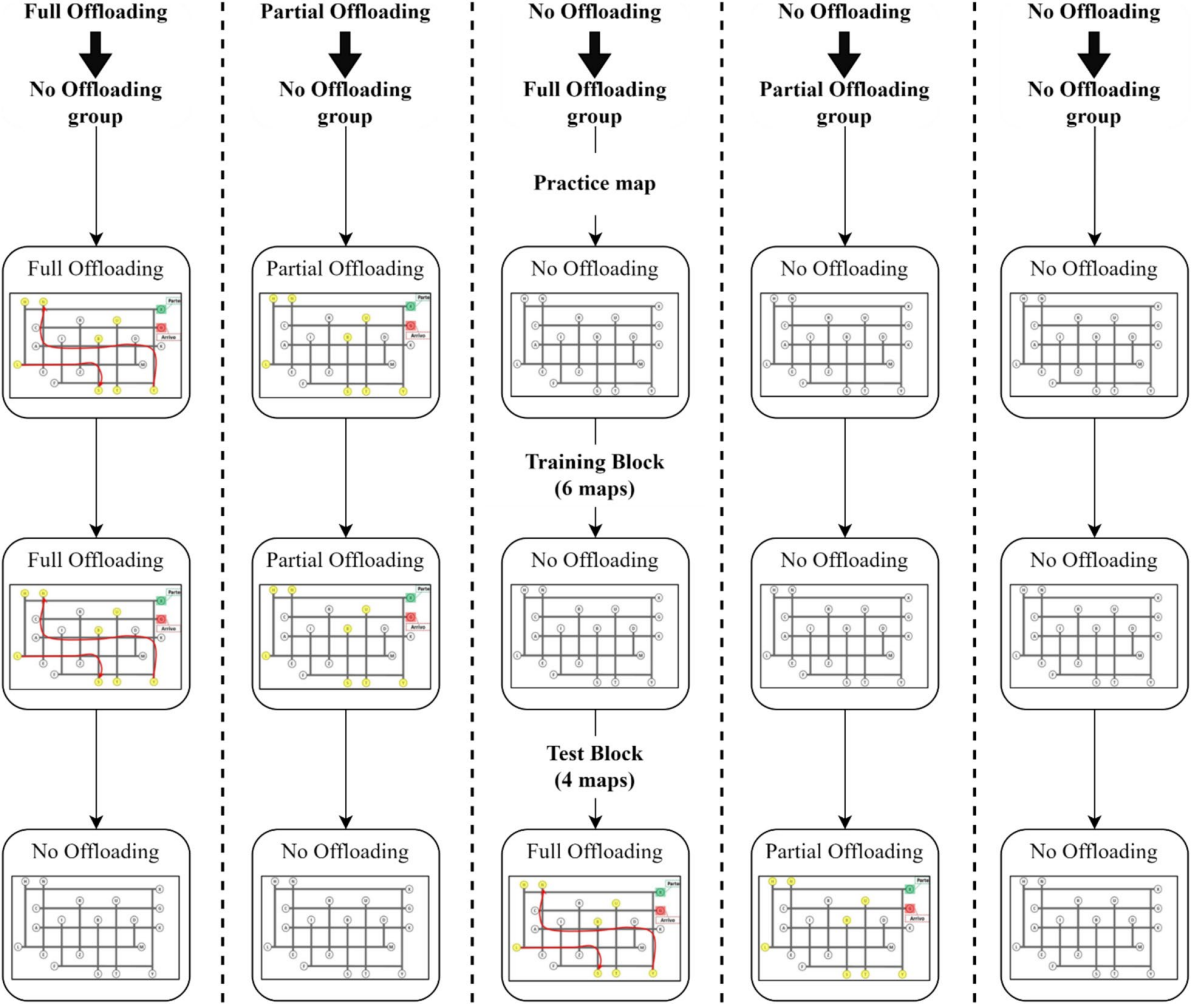
Overview of the Design and Procedure in Experiment 2. *Note*. For clarity, the figure does not show steps related to instructions, performance prediction and postdiction, and evaluation of perceived difficulty and effort (see Sect. "Procedure").

The entire experimental procedure was generated and administered using the Qualtrics XM software (for a graphical summary, see Fig. 7).

Participants first read and approved the informed consent. Then, they read the instructions for the training block of the route planning task, which asked them to plan the shortest route for each map as quickly and accurately as possible. The instructions also specified that, after fully identifying the route for a given map, participants had to click on a “Next” button to access the response frame below the map, where they had to type the locations (letters) in the exact planned order of visit. Then, they had to press another “Next” button to access a new map. Participants were not allowed to return to previously completed steps of the procedure or to previous maps in the training and test blocks.

After the instructions and before starting the training block, participants completed a practice map of the same kind as the ones composing their training block. If they made errors (e.g., incorrect starting/arrival points, or violated ordering rules), they were shown their typed route along with feedback indicating the errors, and they were asked to repeat the practice map. If errors persisted after the second attempt, participants were presented with a correct example of a route that met all task criteria, ensuring they understood task requirements before proceeding to the training block. Then, participants provided performance estimates on route planning accuracy and speed and started the training block.

**Fig. 8:**
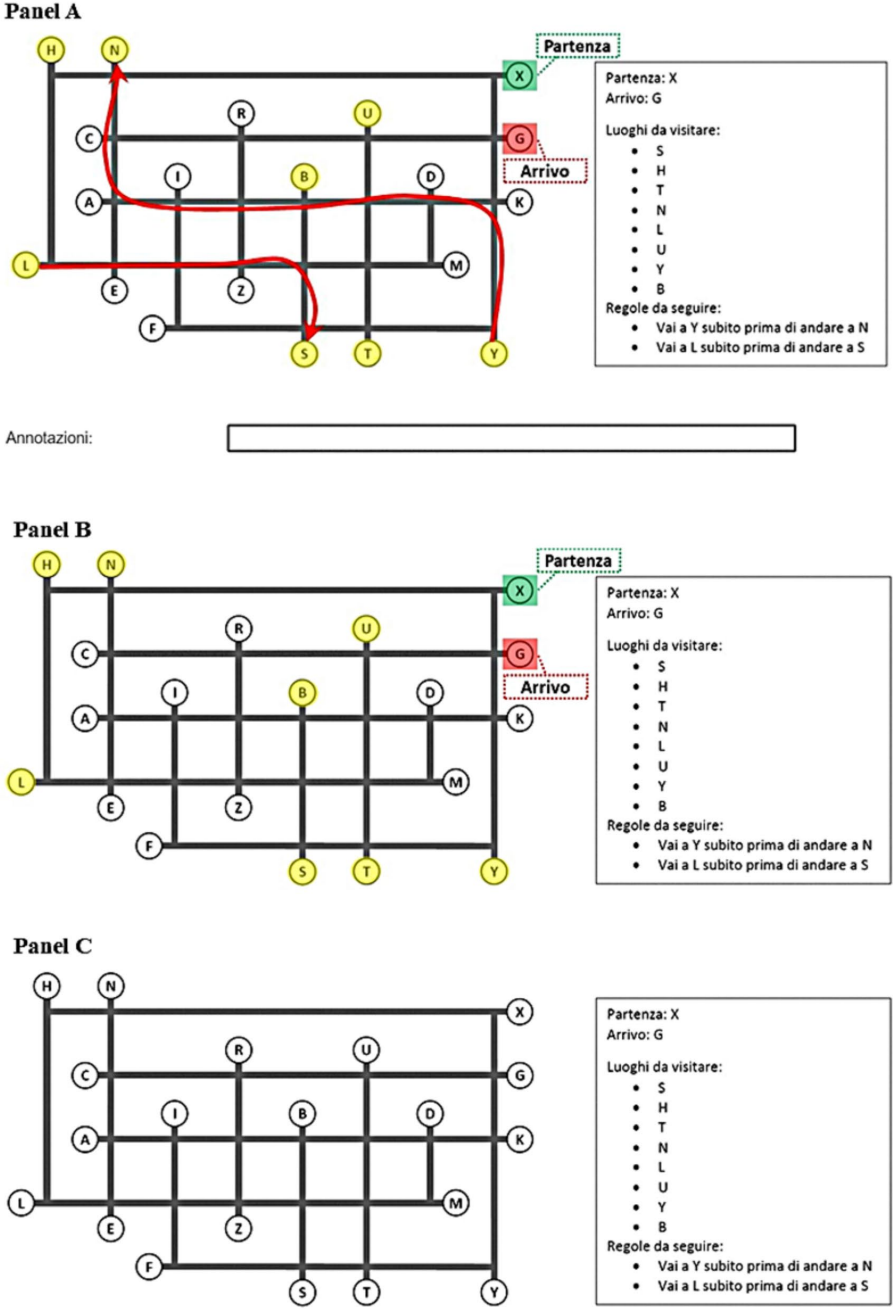
Example Map in the Full Offloading (Panel A), Partial Offloading (Panel B), and No Offloading (Panel C) Conditions in Experiment 2. *Note*. In Panel A, the starting place (X—*Partenza*) and arrival place (G—*Arrivo*) are verbally labeled and visually highlighted in green and red, respectively, within the map. Locations to be visited (S, H, T, N, L, U, Y, B—*Luoghi da visitare*) are highlighted in yellow, and ordering rules (Y-N, L-S—*Regole da seguire*) are represented as directed red arrows. A textbox labeled *Annotazioni* (notes) below the map provides space to track plan progress. In Panel B, only the starting place (X—*Partenza*), arrival place (G—*Arrivo*), and locations to be visited (S, H, T, N, L, U, Y, B—*Luoghi da visitare*) are visually highlighted. In Panel C, no visual aids for offloading strategies are provided

In the training block, participants in the full offloading → no offloading group were shown maps that, along with their specific directions on the right (e.g., starting and ending locations, intermediate locations to be visited, and ordering rules), presented starting/arrival locations and intermediate locations visually highlighted, locations constrained by ordering rules connected by red arrows, and a textbox for tracking their plan (see Fig. 8, panel A). The partial offloading → no offloading group was shown maps with starting/arrival locations and intermediate locations visually highlighted, but no visual aids for ordering rules or plan tracking (see Fig. 8, panel B). Participants in the no offloading → full offloading, no offloading → partial offloading, and no offloading → no offloading groups were shown maps with their specific directions on the right panel only and no visual aids (see Fig. 8, panel C).

After completing the maps in the training block, participants provided retrospective judgments on performance and rated the difficulty and effort required to complete the training block. Next, participants received instructions for the test block. Participants in the no offloading → no offloading group were informed that the test block would be identical to the training block. Those in the other four groups were informed that their task conditions would differ from those in the training block, with specific details provided for each group.

After completing the test block, participants performed the backward digit span task (Monaco et al., 2015) and the visual search task (MFTC; Marra et al., 2013). Finally, they answered demographic questions. The entire procedure lasted a median of 49.55 min. A debriefing session was conducted after data collection, during which participants were fully informed of the study aims and additional details were provided.

#### Materials

##### Tasks and measures

A computerized version of the route planning task was created using the Qualtrics XM software. The maps were a subset of those used in Experiment 1, specifically selected based on their comparability in terms of solution speed and accuracy when completed under the no-pen condition. The training block consisted of 6 maps as in Experiment 1, but only one test block, consisting of 4 maps, was presented. This change was aimed at simplifying the procedure and reducing the session length, thereby minimizing participants’ fatigue and potentially increasing task compliance in our online setting. The maps assigned to the training block were comparable to those in the test block.

As in Experiment 1, for error-free maps we assessed planning accuracy by the proportional deviation above the shortest route and map completion time by the time interval from the map presentation to participants’ click on the “Next” button to move to the following map. Additionally, the time spent typing the planned route was measured by recording the time interval between participant’s access to the response frame after having planned the route and the “Next” button click to access to the following map.

Participants completed a computerized version of the Backward Digit Span task adapted from Monaco et al. (2015). In this task, participants were presented with a sequence of digits, each displayed on the screen for one second. The initial span started with two digits. After viewing the sequence, participants were required to type the digits in reverse order. If they responded correctly, the digit span increased by one in the next trial. If they made an error, a new sequence of the same length was presented. Participants were given two attempts to correctly recall a sequence for each span length. If they failed twice at the same span length, their span score corresponded to the maximum number of digits correctly recalled in reverse order, with a maximum possible score of 9. To assess visual search ability, we used the MFTC (Marra et al., 2013) as in Experiment 1.

All data analyses were conducted using Jamovi (version 2.6.44), except for specific planned comparisons, which were conducted in R (version 4.4.1).^9^

### Results

#### Preliminary analyses

For each performance variable of the route planning task, data were trimmed within groups of participants and blocks of maps. Values that were 3 *SD*s above or below the mean were replaced by the mean ± 3 *SD*s, as in Experiment 1. For verbal working memory and visual search measures, the trimming procedure was applied on the entire sample. Descriptive statistics for all route planning task measures by groups and blocks are presented in Table 2.^10^

**Table 2:** Descriptive statistics of route planning performance measures in experiment 2

Block	Proportional deviation above the shortest route		Map completion time		Solution-typing time		Perceived task difficulty		Perceived task effort	
	Training	Test	Training	Test	Training	Test	Training	Test	Training	Test
	M (SD)	M (SD)	M (SD)	M (SD)	M (SD)	M (SD)	M (SD)	M (SD)	M (SD)	M (SD)
*GROUP*										
Full → no offloading	0.09	0.18	145.99	266.57	44.06	90.06	4.00	5.41	4.26	5.74
	(0.05)	(0.15)	(59.86)	(109.26)	(26.98)	(57.47)	(0.78)	(1.26)	(1.05)	(1.21)
Partial → no offloading	0.10	0.18	140.57	253.11	56.63	85.95	4.00	5.12	4.36	5.24
	(0.05)	(0.14)	(64.81)	(102.84)	(35.64)	(71.10)	(1.06)	(1.36)	(1.17)	(1.15)
No → full offloading	0.11	0.09	204.03	141.66	54.92	34.65	4.29	2.82	4.68	2.65
	(0.05)	(0.06)	(80.10)	(53.22)	(46.89)	(16.38)	(0.94)	(1.06)	(1.20)	(1.32)
No → partial offloading	0.13	0.11	208.78	159.04	54.27	39.34	4.76	3.65	5.06	3.65
	(0.07)	(0.05)	(67.94)	(64.36)	(39.13)	(20.83)	(1.10)	(1.20)	(1.32)	(1.28)
No → no offloading	0.11	0.14	236.32	217.98	64.19	59.23	4.38	4.71	4.84	4.74
	(0.07)	(0.10)	(85.87)	(81.71)	(50.01)	(40.99)	(1.01)	(1.13)	(1.35)	(1.06)

Map completion times and solution-typing times are indicated in seconds

There were no significant differences between groups in gender, χ^2^(4) = 5.55, *p* = .235, or visual search skills, *F*(4,160) = 0.32, *p* = .861. However, significant between-group differences were found for the verbal working memory measure, *F*(4,161) = 3.56, *p* = .008. Tukey’s post-hoc comparisons indicated that, on average, participants in the partial offloading → no offloading group (*M* = 6.97, *SD* = 1.96) had a slightly higher verbal working memory span than those in the full offloading → no offloading group (*M* = 5.56, *SD* = 1.40), *t*(161) = 3.56, *p* = .004, *d* = .87. No other between-group differences were statistically significant (*t*s < 2.68, *p*s > .062). Given this difference in verbal working memory, subsequent analyses were conducted both with and without controlling for individual differences in verbal working memory, supporting the same conclusions. In the next sections, we report the results without controlling for verbal working memory, while in the Supplementary Materials (section S9) we present the results with this variable controlled.

#### Transfer effects on route planning performance

We conducted a series of 5 (full offloading → no offloading *vs*. partial offloading → no offloading *vs*. no offloading → full offloading *vs*. no offloading → partial offloading *vs*. no offloading → no offloading groups) × 2 (training *vs*. test blocks) mixed ANOVAs and planned comparisons to test our hypotheses on performance measures of the route planning task on the maps completed without errors.^11^ As in Experiment 1, when both groups trained with visual aids for offloading strategies exhibited a significant decline in performance after their removal, and both groups trained without aids for offloading strategies showed a significant improvement after their introduction, we computed difference scores on that measure between the training and test blocks to test H3.

##### Proportional deviation above the shortest route

**Fig. 9:**
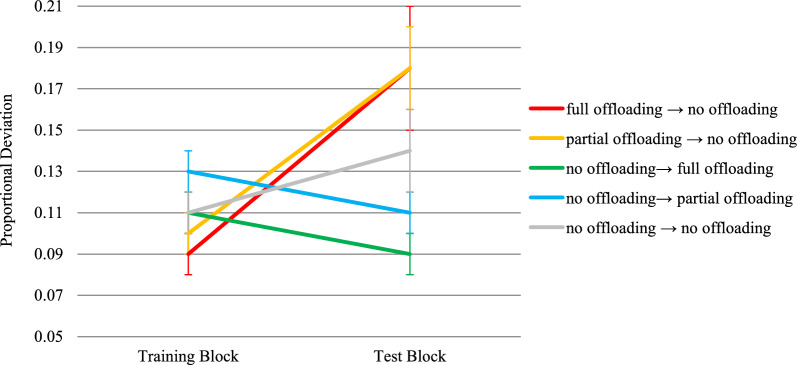
Transfer effects on proportional deviation above the shortest route in experiment 2. *Note*. Bars represent standard errors

The main effect of block on the proportional deviation above the shortest route, *F*(1,158) = 13.61, *p* < .001, η^2^_p_ = .08, and the group × block interaction, *F*(4,158) = 9.89, *p* < .001, η^2^_p_ = .20, were both significant, while the main effect of group was not, *F*(4,158) = 1.48, *p* = .209, η^2^_p_ = .04 (see Fig. 9 and Table 2 for descriptive statistics). In agreement with H1, participants in both the full offloading → no offloading group and the partial offloading → no offloading group planned routes that deviated significantly more from the shortest routes in the test block than in the training block, *t*(158) = 4.78, *p* < .001, *d* = .61 and *t*(158) = 4.74, *p* < .001, *d* = .66, respectively. Conversely, neither the groups provided with full or partial aids for offloading strategies in the test block nor the control group exhibited significant differences in deviation above the shortest routes between the training and test blocks, *t*s <|1.65|, *p*s > .102, in contrast with H2. Although the group means were in the expected direction (see Table 2), the planned comparison contrasting participants in the full offloading → no offloading and in the partial offloading → no offloading groups with participants in the no offloading → no offloading group on their proportional deviation above the shortest route in the test block did not reach significance, *t*(158) = 1.47, *p* = .144, *d* = .26.

Participants in the full offloading → no offloading group did not significantly differ from participants in the partial offloading → no offloading group in their deviation above the shortest route during the training block, *t*(158) = 0.34, *p* = .738, *d* = .04, nor did participants in the no offloading → full offloading group differ significantly from those in the no offloading → partial offloading group in the test block, *t*(158) = 0.82, *p* = .416, *d* = .39. These results supported H7.

##### Map completion time

**Fig. 10:**
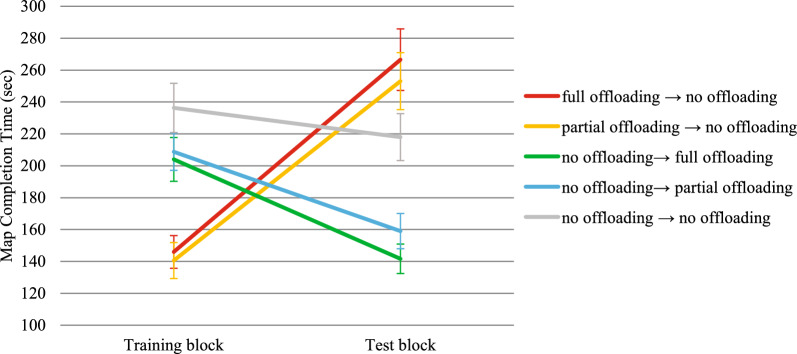
Transfer effects on map completion time in experiment 2. *Note*. Bars represent standard errors

The analysis on map completion time showed significant main effects of the group, *F*(4,158) = 3.27, *p* = .013, η^2^_p_ = 0.08, and of the block, *F*(1,158) = 10.09, *p* = .002, η^2^_p_ = 0.06. The group × block interaction was also significant, *F*(4,158) = 37.53, *p* < .001, η^2^_p_ = 0.49 (see Fig. 10 and Table 2 for descriptive statistics). In agreement with H1, participants in both the full offloading → no offloading and partial offloading → no offloading groups required significantly more time to complete a map in the test block than in the training block, *t*(158) = 8.10, *p* < .001, *d* = 1.19 and *t*(158) = 7.89, *p* < .001, *d* = 1.33, respectively. Conversely, in agreement with H2, participants in the no offloading → full offloading and no offloading → partial offloading groups spent significantly less time completing maps in the test block than in the training block, *t*(158) = − 4.20, *p* < .001, *d* = .94, and *t*(158) = − 3.54, *p* < .001, *d* = .61, respectively. Participants in the no offloading → no offloading group did not exhibit significant differences in map completion time between the training block and the test block, *t*(158) = − 1.25, *p* = .214, *d* = .24.

The planned comparison contrasting participants in the full offloading → no offloading and partial offloading → no offloading groups with participants in the no offloading → no offloading group on map completion time in the test block was statistically significant, *t*(158) = 2.26, *p* = .025, *d* = .42. These results showed that participants in the full offloading → no offloading and partial offloading → no offloading groups were significantly slower in planning their routes in the test block compared to participants in the no offloading → no offloading group, further supporting H3.

In the training block, participants in the full offloading → no offloading group did not complete the task significantly faster than those in the partial offloading → no offloading group, *t*(158) = 0.49, *p* = .627, *d* = .09. Likewise, in the test block, participants in the no offloading → full offloading group were not significantly faster than those in the no offloading → partial offloadin*g* group, *t*(158) = 0.84, *p* = .403, *d* = .29. These results support H7.

To test whether the slowdown in map completion time shown by participants in the full offloading → no offloading and partial offloading → no offloading groups exceeded the speedup by participants in the no offloading → full offloading and no offloading → partial offloading groups (H3), we calculated the difference in map completion time (Δ map completion time) between the training and test blocks for each of these groups.^12^ Δ map completion time was computed as the difference between the block with visual aids and the block without visual aids, with a higher Δ map completion time indicating a greater slowdown after the removal of visual aids or a greater speed up after their introduction, depending on the group.

**Fig. 11:**
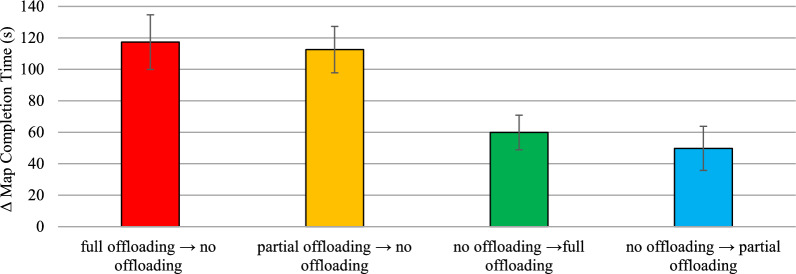
Extent of Slowdown/Speedup in Absolute Value After Removal/Introduction of Visual Aids for Offloading Strategies in the Test Block as a Function of the Group in Experiment 2. *Note*. Bars represent standard errors

A one-way ANOVA on Δ map completion time revealed significant between-group differences, *F*(3,128) = 5.90, *p* < .001, η^2^ₚ = .12. Participants in the full offloading → no offloading group (*M* = 117.30 s, *SD* = 98.29 s) had significantly higher Δ map completion times than participants in both the no offloading → full offloading group (*M* = 59.85 s, *SD* = 63.38 s), *t*(128) = 2.79, *p* = .006, *d* = .69, and in the no offloading → partial offloading group (*M* = 49.74 s, *SD* = 81.78 s), *t*(128) = 3.31, *p* = .001, *d* = .82. Participants in the partial offloading → no offloading group (*M* = 112.54 s, *SD* = 84.78 s) also showed significantly higher Δ map completion times compared to participants in both the no offloading → full offloading group, *t*(128) = 2.58, *p* = .011, *d* = .64, and the no offloading → partial offloading group, *t*(128) = 3.10, *p* = .002, *d* = .76. The remaining between-group comparisons were not statistically significant (*t*s < 0.51, *p*s > .617), further supporting the idea that removing/introducing aids for either two or four offloading strategies did not differentially increase/decrease map completion time (H7). See Fig. 11 for a graphical representation of Δ map completion time as a function of the group.

#### Solution-typing time and verbal working memory

As a preliminary step for testing H4, we appraised the effects of the group and the block on solution-typing time. The mixed ANOVA showed a significant group × block interaction, *F*(4,158) = 16.42, *p* < .001, η^2^_p_ = .29, and significant main effects of group, *F*(4,158) = 3.18, p = .015, η^2^_p_ = .07, and block, *F*(1,158) = 4.80, p = .030, η^2^_p_ = .03 (see Table 2 for descriptive statistics). Consistently with H1, participants in both the full offloading → no offloading and partial offloading → no offloading groups spent significantly more time typing the solution in the test block than in the training block, *t*(158) = 6.36, *p* < .001, *d* = .95, and *t*(158) = 4.09, *p* < .001, *d* = .54, respectively. In agreement with H2, participants in the no offloading → full offloading group spent significantly less time reporting their solutions in the test block than in the training block, *t*(158) = − 2.84, *p* = .005, *d* = .53. Similarly, participants in the no offloading → partial offloading group were faster in typing the solution in the test block than in the training block, *t*(158) = − 2.11, *p* = .036, *d* = .61. Participants in the no offloading → no offloading group did not exhibit significant differences in solution-typing time between the training block and the test block, *t*(158) = − 0.67, *p* = .503, *d* = .15.

According to H4, participants initially trained with external visual aids for offloading strategies should experience a less pronounced slowdown in solution-typing time after the removal of these aids if they had better verbal working memory. To test this hypothesis, we focused on Δ solution-typing time between the training and test blocks, with a higher Δ solution-typing time indicating a greater slowdown for the full offloading → no offloading and partial offloading → no offloading groups after the removal of visual aids, and a greater speedup for the no offloading → full offloading and no offloading → partial offloading groups after their introduction. A General Linear Model (GLM) was used, with group, verbal working memory score (Backward Digit Span), and their interaction as predictors of Δ solution-typing time.^13^ The interaction between group and verbal working memory was statistically significant, *F*(3, 124) = 2.92, *p* = .037, η^2^ₚ = .07, and the group was a significant predictor, *F*(3,124) = 4.11, *p* = .008, η^2^ₚ = .09, while verbal working memory score was not, *F*(1,124) = 2.84, *p* = .094, η^2^ₚ = .02. Simple effects analyses revealed that group differences in Δ solution-typing time were significant when verbal working memory scores were 1 *SD* below the mean, *F*(3,124) = 5.84, *p* < .001, η^2^ₚ = .12, and at the mean, *F*(3,124) = 2.81, *p* = .042, η^2^ₚ = .06, but not when scores were 1 *SD* above the mean, *F*(3,124) = 0.41, *p* = .750, η^2^ₚ = .01. Specifically, when verbal working memory scores were 1 *SD* below the mean, participants in the full offloading → no offloading group had significantly greater Δ solution-typing times than those in the no offloading → full offloading group, *t*(124) = 3.23, *p* = .002, *d* = .80, and the no offloading → partial offloading group, *t*(124) = 3.05, *p* = .003, *d* = .75. No significant difference was found between the full offloading → no offloading and partial offloading → no offloading groups, *t*(124) = 0.05, *p* = .961, *d* = .01.

**Fig. 12:**
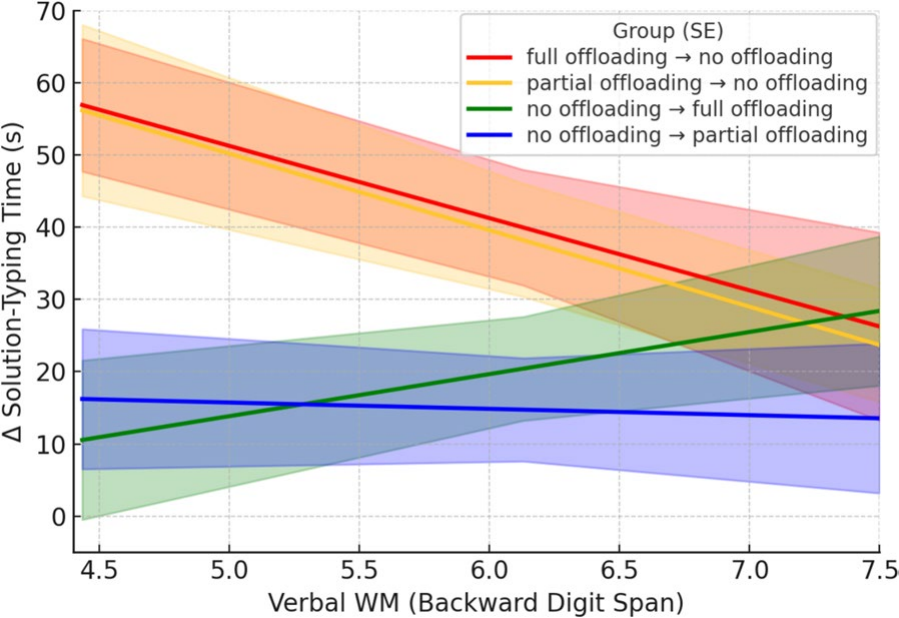
Extent of Slowdown/Speedup in Solution-Typing Time in Absolute Value After Removal/Introduction of Offloading Strategies as a Function of Verbal Working Memory in Experiment 2

At the mean verbal working memory score, participants in the full offloading → no offloading group did not show significantly higher Δ solution-typing times than those in the no offloading → full offloading group, *t*(124) = 1.82, *p* = .072, *d* = .45, but they did exhibit significantly higher Δ solution-typing times than those in the no offloading → partial offloading group, *t*(124) = 2.35, *p* = .020, *d* = .58. No significant differences were found between the full offloading → no offloading and partial offloading → no offloading groups, *t*(124) = 0.16, *p* = .876, *d* = .04. Importantly, when verbal working memory scores were 1 *SD* above the mean no significant between-group differences in Δ solution-typing times were observed (*t*s < 0.55, *p*s > .589) (see Fig. 12). The same pattern of results was observed when the partial offloading → no offloading group was used as the reference group instead of the full offloading → no offloading group (see Supplementary Materials, section S10). These findings agree with H4.

#### Transfer effects on perceived task difficulty and effort

To investigate whether the removal of visual aids for offloading strategies led to an increase in perceived task difficulty and effort and their introduction led to opposite effects, we ran on these variables the same analyses as for transfer effects on performance. We summarize the main results of these analyses here; full details are presented in Supplementary Materials, Section S11 (for descriptive statistics, see Table 2). Consistent with H5, removing visual aids for offloading strategies led to a significant increase in perceived task difficulty and effort (post-hoc comparisons: *t*s > 4.17, *p*s < .001) for participants trained with these aids. Conversely, consistent with H6, introducing visual aids for offloading strategies decreased these perceptions for participants trained without aids (post-hoc comparisons: *t*s > 5.73, *p*s < .001). Additionally, participants in the full offloading → no offloading and partial offloading → no offloading groups rated the test block as more difficult and effortful than those in the no offloading → no offloading group (post-hoc comparisons: *t*s > 2.11, *p*s < .036), highlighting the impact of prior experience with visual aids for offloading on perceived difficulty and effort once these aids were removed. Finally, while participants trained with full or partial aids for offloading strategies did not differ in their perception of task difficulty and effort during the training block (post-hoc comparisons: *t*s < 0.35, *p*s > .732), participants provided with aids in the test block reported greater relief in both perceived task difficulty and effort when given full aids compared to partial aids (post-hoc comparisons: *t*s > 2.80, *p*s < .007).

### Discussion

Participants trained with visual aids for offloading strategies exhibited a worse performance both in planning accuracy and in map solution times when visual aids were removed in the test stage, thus fully supporting H1. Participants trained without visual aids for offloading strategies showed a decrease in map solution times but not a significant increase in planning accuracy when visual aids were introduced in the test stage, thus partially supporting H2. The absence of significant effects for planning accuracy could be possibly explained by the observation that accuracy was already good in the training stage even without visual aids. Participants trained with visual aids for offloading strategies showed a significantly greater slowdown in map completion time compared to the speedup observed in participants who were introduced to these aids during the test stage, thus supporting H3. The asymmetric transfer effect on solution-typing time was evident for participants with lower verbal working memory and, partially, for those with average verbal working memory, but not for participants with higher verbal working memory, in line with the expected moderation effect (H4). As expected, the removal of visual aids in the test stage increased perceived task difficulty and effort (H5) while their introduction reduced these perceptions (H6). Finally, no significant performance differences were observed between participants provided with visual aids for all four offloading strategies and those provided with only two aids in either the training or the test block (H7). However, providing four (vs. two) aids in the test block resulted in a greater relief in terms of perceived task difficulty and effort.

The findings on planning accuracy and time obtained in Experiment 2 closely mirrored the ones obtained in Experiment 1, despite the significant change in the way offloading strategies were supported: allowing the use of a pen during route planning vs. embedding specific visual aids into the maps. This confirms the positive effects on route planning performance of the opportunity to offload cognition and the negative effects of the removal of this opportunity. The results of Experiment 2 are also consistent with the idea that abandoning a visual strategy in favor of a strategy based on working memory is more challenging than the opposite shift, requiring a deeper reconfiguration and suffering from a greater interference from the strategy previously employed. The outcomes of Experiment 2 show that opposite switches between distinct strategies can lead to different cognitive consequences. The moderation effect by individual differences in working memory strengthens this interpretation.

Participants provided with aids for all four offloading strategies did not outperform those with aids for only two strategies in either the training or test block. This finding aligns with results from Experiment 1, in which participants trained using the pen employed more offloading strategies during the test block compared to those who were trained without it but with no performance benefits.

Finally, in terms of task perception, the removal of visual aids for offloading strategies led to an increase in perceived task difficulty and effort for participants trained with these aids, while the introduction of visual aids had the opposite effect, suggesting that prior (*vs*. no prior) experience with cognitive offloading not only hindered task performance when offloading became unavailable, but also affected task perception. Additionally, participants provided with four visual aids in the test block perceived a greater reduction in both task difficulty and effort compared to those given only two, possibly reflecting a greater subjective relief when more external support was offered.

## General discussion

The primary aim of our experiments was to investigate the bidirectional transfer effects of cognitive offloading in a complex task such as route planning, specifically examining how the opportunity to offload or not offload cognition during a route planning task affected subsequent performance when this option was either removed or introduced. By exploring these shifts, we aimed to extend the current understanding of the consequences of cognitive offloading, providing, to our knowledge, the first insights into how these bidirectional transfer effects take place in a route planning task. In Experiment 1, following Florean et al. (2025a), participants freely devised their own offloading strategies using a pen while completing the task, while in Experiment 2 visual aids were embedded into the maps to support these offloading strategies.

In agreement with our hypothesis (for a summary, see Table 3), the results of both experiments showed that removing the opportunity to offload cognition after a stage in which this opportunity was allowed negatively affected both planning accuracy and planning time (H1). Introducing this opportunity after a stage in which it was precluded reduced planning time but did not always significantly affect planning accuracy (H2), possibly because accuracy was already high and the room for improvement was small. Both experiments exposed the expected asymmetric transfer effects on route planning performance (H3), showing that participants trained with the opportunity to offload cognition experienced a greater increase in map completion time when this opportunity was removed compared to the decrease observed when the opportunity to offload cognition was given to participants initially trained without it. The asymmetric transfer effect on map completion time was moderated by individual differences in spatial working memory in Experiment 1, while the asymmetric transfer effect on solution-typing time was moderated by individual differences in verbal working memory in Experiment 2, in agreement with the hypothesis that working memory capacity is relevant to adapt to the removal of the opportunity to offload cognition in our route planning task. Finally, in both the experiments, the results supported the hypotheses that removing offloading opportunity increases perceived task difficulty and effort (H5), while introducing such opportunity reduces perceived task difficulty and effort (H6), in line with previous findings (Florean et al., 2025a).

**Table 3:** Summary of the hypotheses and support provided by experiment 1 and 2

Hypothesis	Prediction	Support
H1	Removing offloading opportunity reduces planning accuracy and increases map solution times	Support for planning accuracy (Exp. 1 and 2) Support for map solution times (Exp. 1 and 2)
H2	Introducing offloading opportunity improves planning accuracy and decreases map solution times	Partial support for planning accuracy (Exp. 1) Support for map solution times (Exp. 1 and 2)
H3	The effect of offloading removal exceeds the effect of offloading introduction (asymmetric transfer effect)	Partial support for planning accuracy (Exp. 1) Support for map solution times (Exp. 1 and 2)
H4	The asymmetric transfer effect is moderated by individual differences in working memory capacity	Support for map solution times (Exp. 1) Support for solution-typing times (Exp. 2)
H5	Removing offloading opportunity increases perceived task difficulty and effort	Support for difficulty (Exp. 1 and 2) Support for effort (Exp 1 and 2)
H6	Introducing offloading opportunity decreases perceived task difficulty and effort	Support for difficulty (Exp. 1 and 2) Support for effort (Exp 1 and Exp 2)
H7	No differences in performance of the removal/introduction of 4 vs. 2 visual aids embedded in maps as a support for offloading strategies	Support (Exp. 2)

### Theoretical implications

These results significantly extend the body of work on the positive and negative effects of cognitive offloading (e.g., Eskritt & Ma, 2014; Fenech, 2010; Henkel, 2014; Sparrow, 2011; for reviews, see Gilbert et al., 2023, Gilbert, 2024; Risko & Gilbert, 2016) by providing specific insight into transfer-related consequences. On the one hand, they show that offloading supports immediate performance even when the opportunity to offload cognition is introduced after a stage in which participants were initially not offered such an opportunity. Participants can quickly learn how to offload cognition in our task when allowed to do so, despite a previous experience with the task when offloading was precluded. The benefits of offloading were observed both when participants were free to devise their strategies and when offloading was supported by visual aids. On the other hand, our results showed that removing the opportunity to offload cognition after practicing the task with such an opportunity had detrimental effects that are greater, in absolute value, than the positive effects observed in the opposite condition. The asymmetric transfer effects observed in both experiments agree with our expectation that shifting from a visual strategy to a strategy based on working memory for route planning when offloading is precluded is more challenging than the reverse shift when offloading is allowed. The involvement of working memory in the adaptation to the former shift was supported by the significant buffering role of individual differences in working memory.

Overall, findings on the measures of task perception in Experiments 1 and 2 agree with previous accounts in the literature on cognitive offloading, which suggest that the avoidance of cognitive effort may be a key factor driving the tendency to offload cognition (Gilbert et al., 2023; Sachdeva & Gilbert, 2020; see also Florean et al., 2025a), and in our case it may even contribute to explain the asymmetric transfer effects. Indeed, when the opportunity for offloading was removed, participants may have unsuccessfully attempted to use or adapt the intuitive and less effortful visual strategies they previously employed, and this may have hindered the adoption of the cognitively demanding incremental planning strategy.

The results showing that individual differences in working memory moderated asymmetric transfer effects suggest that working memory eased the transition to strategies requiring internal cognitive resources of participants initially trained with the opportunity to offload cognition. Thus, a greater working memory span seems to attenuate the negative consequences of a strategy shift between an externally aided and an internally driven strategy, an interesting aspect that warrants further investigation.

These results offer a more complex and articulate picture than the ones that can be reached starting from the literature on transfer effects in problem solving that did not consider the effects of the opportunity to offload cognition, such as the studies on negative transfer of previously learned strategies (Bilalić & McLeod, 2014; Bilalić et al., 2008; Luchins & Luchins, 1950; Ohlsson, 2011), and the studies on the positive consequences of learning to perform a task with a planful strategy triggered by high operator cost when the cost of operators is subsequently reduced (O’Hara & Payne, 1998). Participants who were trained with the opportunity to offload cognition struggled more to abandon their previous strategy, which was challenging to replace due to its reliance on intuitive visual routines (see, e.g., MacGregor & Chu, 2011; MacGregor & Ormerod, 1996; Vickers et al., 2001). In contrast, participants trained without the option to offload cognition adapted more readily when offloading was introduced, as this allowed them to switch from a cognitively demanding planning strategy (e.g., Wiener et al., 2009) to a visual strategy that was more intuitive, less cognitively demanding, and thus easier to adopt. These results indicate that the ease or difficulty of the shift between planning strategies is contingent upon the cognitive features of both the initial and final strategies. More broadly, our results highlight the importance of considering the specific interaction between the strategies used by participants over time. Further research should examine when these asymmetric transfer effects occur across different types of cognitive tasks and offloading strategies.

Reconciling our results with previous studies on transfer in planning and problem solving requires to better specify the conditions in which previous experience on a task facilitate or hinder subsequent performance in the same task. Previous research has shown benefits when the strategies used in the training and test stage are the same or at least they share some of their fundamental routines (e.g., Anderson, 1993) or when the initial practice on the task during training provides insight on how to solve it in the transfer stage (O’Hara & Payne, 1998). Positive transfer effects to different tasks (e.g., O’Hara & Payne, 1998; Singley & Anderson, 1989) or even to the same task presented in an apparently different form (e.g., Gick & Holyoak, 1987) are difficult to achieve. When participants need to change the strategy they used in the past for the same task, and the old strategy hinders the development of the new one, then negative transfer effects are usually observed (Luchins & Luchins, 1950; Ohlsson, 2011). Offloading in a planning or problem-solving tasks may change the nature of the task (Norman, 1991) and the strategies applied by participants (Florean et al., 2025a), who usually take advantage from the opportunity to offload cognition to reduce cognitive effort and improve performance (Gilbert, 2024; Risko & Gilbert, 2016). This may imply negative transfer effects when practice with the older strategy, relying on offloading, creates a mental set that is difficult to abandon when the opportunity for offloading is no longer available and when the older strategy interferes with the development of the new one, thus imposing higher reconfiguration costs (Lemaire & Lecacheur, 2010). This can happen, for instance, when offloading promotes the use of a simpler and more effective strategy that cannot be transferred to the same task when offloading is no more available and thus a more cognitively demanding strategy needs to be developed from scratch, as in our experiments (see also Kosmyna et al., 2025). Indeed, according to Lemaire and Lecacheur (2010), once a problem has been solved with a given strategy, the cognitive system must be reconfigured to adopt a different strategy and this requires shifting attention to the new strategy, retrieve associated task goals, rules, or procedures, and apply executive control for activating, executing, and ordering these components while inhibiting irrelevant past strategies. More generally, as O’Hara and Payne (1998) state “…problem solving behavior is inextricably linked with a network of costs and benefits which extends beyond task and solver characteristics into the physical world in which the problem solving is taking place… Thus, when theorizing about problem solving, it is essential to examine the nature of the cost–benefit structure within which the individual and task characteristics are placed” (p. 68). Our experiments show that the opportunity to offload cognition can affect this cost–benefit structure (see also Gilbert, 2024), promoting or hindering the use of specific strategies also in relation with the strategies that have been previously employed.

### Applied implications

The results of our study suggest some potential applied implications. First, they indicate that individuals may need more support when they must develop a strategy based on internal cognitive resources after a period in which they have deeply relied on external aids, tools, and devices (e.g., Brügger et al., 2019; Kosmyna et al., 2025). For instance, individuals who usually rely on a navigation system to plan routes, may get into trouble when the technology is no longer available, and they must use a physical map. Providing indications on how to plan a route on a map, perhaps by using the low-tech offloading strategies we described or by employing cluster/region-based incremental planning, may turn out to be useful. An alternative approach could be to support the human-centered design of less automated route planning systems actively promoting cognitive map learning, considering the potential cognitive drawbacks of fully automated systems (Brügger et al., 2019). This may also facilitate the shift to internal strategies when technologies are not available.

Second, our study, consistent with a previous one (Florean et al., 2025a), identified four effective offloading strategies for route planning spontaneously used by participants. These strategies may potentially inspire the design of training or rehabilitation programs for neuropsychological patients with deficits in spatial planning, a possibility worth exploring in further studies. Furthermore, in Experiment 2, we showed that the incorporation of visual aids supporting these strategies, and in particular location highlighting, significantly improves planning performance in comparison with a condition in which performance is unaided (see also Florean et al., 2025b, for a replication of these results with a real map). These findings suggest that user-centered visual aids for offloading might be useful to support specific populations (e.g., Basso et al., 2001) or even to improve path planning and visualization in navigation systems supporting interactive route planning (see also Florean et al., 2025a).

Third, we provided preliminary evidence for the viability of a user-centered approach based on a reverse engineering approach to cognitive offloading. The approach comprises four steps: (1) observe which strategies participants spontaneously use when allowed to offload cognition to perform a task, (2) appraise their effectiveness, (3) design aids or procedures to support the effective strategies, and (4) empirically test of these aids. More generally, Experiment 1 showed that the availability of a tool (the pen) enables the use of effective offloading strategies (see also Florean et al., 2025a), and Experiment 2 showed that the design of a tool (the map with or without visual aids) can do the same (see also Florean et al., 2025b). This suggests that tools and their specific design can represent physical, sensory, and cognitive affordances for offloading (Hartson, 2003; Norman, 1999). This reconnects offloading research with other streams of research that have examined how cognition stems from a dynamic transaction between the person and the environment in the accomplishment of a task, focusing on interactions between persons and the design of cognitive artifacts (Gray et al., 2006; Guthrie et al., 2015; Hutchins, 1995; Lave, 1988; Norman, 1991).

### Limitations and future research directions

The use of artificially created maps and the recruitment of samples of undergraduate students can be considered as limitations of our experiments, restricting the generalizability of our findings. However, the positive effects on performance of embedding visual aids for offloading into the maps have been replicated with a real-world map of a suburb of Berlin with two different non-German samples taken from undergraduate and general populations (Florean et al., 2025b), suggesting that our findings may generalize to more realistic maps. Further conceptual replications of the present research with different maps and samples could corroborate this evidence, as well as extend the investigation to other age groups, with particular attention to older populations. Evidence suggests that older adults might underutilize offloading strategies due to an overestimation of their unaided abilities (Scarampi & Gilbert, 2021). Yet, it is in this age group that offloading aids may be particularly beneficial, as they can offer key support for daily functioning and autonomy (e.g., Benge & Scullin, 2025).

Another limitation of our study is related to the need to bind the number of experimental conditions and groups in both experiments to allow the proper implementation of complex designs and procedures. Indeed, in Experiment 1 we ensured experimental control by always introducing training-consistent blocks in the test sessions, but we could have additionally added control groups that consistently completed the training and test stages of the task with or without cognitive offloading. Experiment 2 addressed this limitation by including a control group that completed both the training and test stages without offloading, but it did not include a control group that performed the task with visual aids for offloading strategies available from the training to the test stages. Future studies may include both control groups to obtain further information on transfer effects related to cognitive offloading. An additional potential limitation concerns the statistical power of some analyses: the working memory moderation tests (see Section "Map Completion Time" for Experiment 1 and Section "Solution-typing time and verbal working memory" for Experiment 2), the ANCOVA (Supplementary Materials, section S3), and the correlational analyses (Supplementary Materials, section S1). Replicating these findings with larger sample sizes would strengthen our conclusions regarding these analyses.

Experiment 2 was conducted online and thus it did not allow to control for the use of offloading strategies beyond those designed experimentally (e.g., offloading cognition on other devices) or to control for the use of offloading strategies in experimental conditions designed to preclude them (e.g., taking notes on a piece of paper or other devices during planning). However, these concerns should be greatly reduced by the observation that the results of Experiment 2 are largely consistent with those obtained in Experiment 1 (see Table 3), where full experimental control was ensured by the laboratory setting. Moreover, the observed effects of the training group on solution-typing time in Experiment 2 would not have occurred if participants without visual aids had taken notes on the planned path on paper or other devices.

Finally, the results of our experiments should also be considered in the light of our specific experimental conditions, consisting of relatively short training and test sessions, absence of penalties or incentives on performance, focus on short-term transfer effects, and the adoption of a visuospatial planning task. Changes to these aspects of our experimental setting may lead to different results, but we see this more as an opportunity for future research than as a limitation, as further studies with different conditions may help us to better understand the complex cost–benefit structure of offloading-supported planning.

## Conclusion

We investigated the transfer-related consequences of cognitive offloading in a route planning task, showing that the negative effect of removing offloading exceeded the positive effect of its introduction. These findings supported the hypothesis that the transition from a simpler and more intuitive visual planning strategy to a more demanding incremental planning strategy entails higher strategy switching costs and challenges reconfiguration processes more than the reverse transition. More generally, the results show that the opportunity to offload cognition in a complex planning task can affect the strategies used by participants and that this can have cognitive consequences for the subsequent adoption of different strategies when environmental conditions change.

## Significance statement

The manuscript reports on two experiments in which we investigated the transfer-related consequences of cognitive offloading in a spatial planning task similar to the real-world ones. Transfer designs were used to assess the effects of training with or without the opportunity to offload cognition on subsequent planning performance when this opportunity was removed or offered. In Experiment 1, participants were either allowed to use the pen while planning on the map and spontaneously devise offloading strategies or they were prevented from doing so. In Experiment 2, visual aids supporting the offloading strategies observed in Experiment 1 were embedded in the maps in some conditions and not in others. The results of both experiments showed that the negative effect of offloading removal exceeded the positive effect of its introduction. This supports the hypothesis that the transition from a simpler and more intuitive visual planning strategy to a more demanding incremental planning strategy entails higher strategy switching costs and challenges reconfiguration processes more than the reverse transition. More generally, the results show that the opportunity to offload cognition in complex tasks can affect the planning strategies used by participants and their performance. This can have cognitive consequences for the subsequent adoption of different strategies when environmental conditions change. These findings contribute to our understanding of how complex cognition can be supported or hindered by cognitive offloading, an important issue in the context of our society.

## Supplementary Information

Additional file 1.

## Data Availability

Dataset and materials of the experiments are available at the Open Science Framework repository: https://osf.io/h9dwy/?view_only=7e1f2f4f3cb849bc8e3e6379a413a41a.

## Footnotes

1 The travelling salesperson problem requires participants to find the shortest possible route that connects a set of points, visiting each of them only once (e.g., MacGregor, & Chu, 2011).

2 Florean et al. (2025a) obtained a medium effect size for the difference in proportional deviation above the shortest path between participants in the offloading and in the no offloading conditions in high-difficulty maps (Cohen’s *d* = .584; Cohen, 1988).

3 Considering that metacognitive judgments are not relevant to the aims of the study presented here, we do not report the findings related to these measures.

4 In Florean et al. (2025a), two trained raters independently examined 20% of the maps completed in the offloading condition and classified the strategies used. Inter-rater agreement was very high (99%), with a Cohen’s K of .98.

5 To assess visual search ability, we used a computerized version of the Multiple Features Target Cancellation task (MFTC; Marra et al., 2013). Participants were shown a grid of 80 small squares, each containing two differently oriented lines. They were first asked to fixate a probe (a small square with two oriented lines) placed just above the grid and then to identify which of the 80 squares were identical to the probe (13 targets) by clicking on them as quickly and accurately as possible. Timing started when participants, having read the instructions, entered the screen displaying the target and distractor stimuli, and it stopped when participants pressed a bottom-right arrow on the screen to confirm task completion. The accuracy score was calculated according to the formula indicated by Marra et al., i.e., {(correct answers / 13) + [(67 – false alarms) / 67]} /2. Results are reported in Supplementary Materials (sections S1).

6 Data trimming resulted in small percentages of value substitution, ranging from 0 to 3.03%, depending on the condition and block, with no between-group differences in the proportion of substituted values (see Supplementary Materials, section S2). For visual search (MFTC accuracy scores), trimming led to 2.92% substitutions. For spatial working memory (Symmetry Span partial scores), there were no substitutions. Descriptive statistics for the MFTC accuracy score were as follows: *M* = 0.92, *SD* = 0.09. Descriptive statistics for the Symmetry Span task were as follows: *M* = 26.60, *SD* = 7.63.

7 The proportion of maps completed without errors was very high, ranging from 0.84 in training-inconsistent blocks to 0.95 in training-consistent blocks, and from 0.80 in the no-hand → pen group to 0.95 in other groups.

8 Two participants (1.2%) chose not to indicate their gender and seven participants (4.2%) chose not to indicate their age.

9 The planned comparisons contrasting the full offloading → no offloading and partial offloading → no offloading groups with the no offloading → no offloading group were conducted using the afex and emmeans packages in R.

10 Data trimming resulted in rather small percentages of value substitution, ranging from 0 to 5.88% depending on the condition and block, with no between-group differences in the proportion of substituted values (see Supplementary Materials, section S8). For verbal working memory (Backward Digit Span), there were no substitutions (0.00%). For visual search (MFTC accuracy scores), there were also no substitutions (0.00%). One participant (0.01%), specifically from the no offloading → no offloading group, did not complete the Backward Digit Span task. Two participants (0.01%), one from the partial offloading → no offloading group and one from the no offloading → no offloading group, did not complete the MFTC task. Descriptive statistics for the Backward Digit Span were as follows: *M* = 6.09, *SD* = 1.67. Descriptive statistics for the MFTC accuracy score were as follows: *M* = 0.83, *SD* = 0.14.

11 The proportion of maps completed without errors was high, ranging from 0.74 (full offloading → no offloading) to 0.93 (no offloading → partial offloading) in test blocks.

12 The no offloading → no offloading group was not included in this analysis, as it was not relevant for testing H3. As expected, participants in this group did not exhibit significant differences in map completion times between the training and test blocks.

13 The no offloading → no offloading group was not included in this analysis as it was not relevant for testing H4. As expected, participants in this group did not show significant differences in typing-solution times between the training and test blocks.
