# Supplementary material for: Get the unbalance right: asymmetric transfer effects in cognitive offloading
Source: Cogn Res Princ Implic. 2026 Mar 26;11:29. doi: 10.1186/s41235-026-00722-0 (PMC13022156; doi:10.1186/s41235-026-00722-0)
Supplement: Supplementary file 1 — Additional file 1. [file 41235_2026_722_MOESM1_ESM.docx]

**Supplementary Materials for**

**Get the Unbalance Right: Asymmetric Transfer Effects in Cognitive Offloading**

**S1. Experiment 1: Exploratory Analyses on Visual Search, Spatial Working Memory and Route Planning Performance**

We included the Multiple Features Target Cancellation task (MFTC; Marra et al., 2013) as a measure of visual search ability to assess whether a pattern of correlations with route planning measures similar to that observed by Florean et al. (2025) would also emerge in Experiment 1. In that study, when pen use was allowed (i.e., offloading condition) better visual search ability was associated with a greater use of offloading strategies and with a smaller deviation above the shortest route (higher planning accuracy). Additionally, a greater use of offloading strategies was related to better planning accuracy. By contrast, when pen use was precluded (i.e., in the no-offloading condition), better spatial working memory was associated with higher planning accuracy. These findings supported the idea that participants relied on a primarily visual strategy when the opportunity to offload cognition was available, whereas they engaged in a working memory-based strategy when offloading was not available.

In Experiment 1, we conducted correlation analyses separately for the condition allowing pen use and for the conditions precluding pen use (i.e., no-pen and no-hand), as offloading availability (rather than group assignment) was expected to determine the strategy employed. Analyses were performed separately for the training and test stages. When pen use was allowed, better visual search ability was positively correlated with the use of offloading strategies in the training stage, *r*(69) = .32, *p* = .006, and a greater use of offloading strategies was associated with higher planning accuracy in the test stage, *r*(135) = –.23, *p* = .007. Conversely, when pen use was precluded, better spatial working memory was associated with higher planning accuracy both in the training stage, *r*(64) = –.30, *p* = .016, and in the test stage, *r*(133) = –.24, *p* = .004.

This pattern of results is consistent with Florean et al. (2025) and it provides further support for the idea that the availability of external supports for offloading not only reduces cognitive load but also changes the type of cognitive strategies employed, shifting reliance from internal working memory resources to more visually based planning strategies. However, given the relatively modest sample size available for this set of correlation analyses, their findings should be interpreted cautiously.

**S2. Experiment 1: Outlier Detection and Exploratory Analysis on Group Differences in Outlier Substitution**

To address potential concerns regarding the impact of extreme values on the analyses of Experiment 1, we applied a systematic outlier identification and trimming procedure to the raw data on performance measures from the route planning task. For each performance measure, outliers were defined as observations falling outside ± 3 *SD* from the group-specific mean. This criterion was applied separately for each group and block (Del Missier et al., 2021; Florean et al., 2025; Miyake et al., 2000). The performance measures considered were the proportional deviation above the shortest route and map completion time. Group-specific descriptive statistics on the raw (untrimmed) data, including means, standard deviations, and corresponding *M*s ± 3 *SD*s thresholds, are reported in Tables S1-S2.

**Table S1**

*Descriptive Statistics for Raw Data and Percentages of Outlier Substitution for Proportional Deviation Above the Shortest Route in Experiment 1*

|  | **Training Block** | | | | **Training-Consistent Test Block** | | | | **Training-Inconsistent Test Block** | | | |
| --- | --- | --- | --- | --- | --- | --- | --- | --- | --- | --- | --- | --- |
|  |  | Thresholds | |  |  | Thresholds | |  |  | Thresholds | |  |
| **Group** | *M*  (*SD*) | Lower (*M* – 3 *SD*) | Upper (*M* + 3 *SD*) | % Outlier Substitution | *M*  (*SD*) | Lower (*M* – 3 *SD*) | Upper (*M* + 3 *SD*) | % Outlier Substitution | *M*  (*SD*) | Lower (*M* – 3 *SD*) | Upper (*M* + 3 *SD*) | % Outlier Substitution |
| **pen→no-pen** | 0.12 (0.06) | -0.06 | 0.30 | 0.00% | 0.10 (0.09) | -0.17 | 0.37 | 2.86% | 0.16 (0.13) | -0.23 | 0.55 | 2.94% |
| **pen→no-hand** | 0.10 (0.05) | -0.05 | 0.25 | 0.00% | 0.11 (0.10) | -0.19 | 0.41 | 2.78% | 0.18 (0.14) | -0.24 | 0.60 | 0.00% |
| **no-pen→pen** | 0.13 (0.09) | -0.14 | 0.40 | 3.03% | 0.16 (0.16) | -0.32 | 0.64 | 0.00% | 0.12 (0.09) | -0.15 | 0.39 | 3.03% |
| **no-hand→pen** | 0.13 (0.10) | -0.17 | 0.43 | 0.00% | 0.16 (0.14) | -0.26 | 0.58 | 0.00% | 0.12 (0.12) | -0.24 | 0.48 | 3.03% |

**Table S2**

*Descriptive Statistics for Raw Data and Percentages of Outlier Substitution for Map Completion Time in Experiment 1*

|  | **Training Block** | | | | **Training-Consistent Test Block** | | | | | **Training-Inconsistent Test Block** | | | | | | |
| --- | --- | --- | --- | --- | --- | --- | --- | --- | --- | --- | --- | --- | --- | --- | --- | --- |
|  |  | Thresholds | |  |  | Thresholds | | |  | |  | | Thresholds | | |  |
| **Group** | *M*  (*SD*) | Lower (*M* - 3 *SD*) | Upper (*M* + 3 *SD*) | % Outlier Substitution | M  (*SD*) | Lower (*M* – 3 *SD*) | Upper (*M* + 3 *SD*) | % Outlier Substitution | | | | *M* (*SD*) | | Lower (*M* – 3 *SD*) | Upper (*M* + 3 *SD*) | % Outlier Substitution |
| **pen→no-pen** | 188.17 (67.31) | -13.76 | 390.10 | 2.86% | 162.34 (51.12) | 8.98 | 315.70 | 0.00% | | | | 238.73 (70.32) | | 27.77 | 449.69 | 2.94% |
| **pen→no-hand** | 197.59 (55.35) | 31.54 | 363.64 | 2.78% | 164.49 (51.78) | 9.15 | 319.83 | 0.00% | | | | 231.42 (85.73) | | -25.77 | 488.61 | 0.00% |
| **no-pen→pen** | 227.30 (72.68) | 9.26 | 445.34 | 3.03% | 205.22 (57.26) | 33.44 | 377.00 | 0.00% | | | | 179.01 (42.65) | | 51.06 | 306.96 | 3.03% |
| **no-hand→pen** | 203.41 (65.43) | 7.12 | 399.70 | 3.03% | 204.13 (77.51) | -28.40 | 436.66 | 0.00% | | | | 173.94 (51.98) | | 18.00 | 329.88 | 3.03% |

*Note.* Mean (*M*), Standard Deviation (*SD*), and Threshold values are expressed in seconds.

As the number of outliers per cell was too small for conducting a chi-square test, between-group differences in outlier substitution were assessed by conducting *z* tests for differences in proportions. Specifically, using the most conservative procedure, we compared the frequency of outliers between the two groups showing the highest and lowest proportions of outliers for each measure and block. Test statistics are reported in Table S3. No significant differences between the two most-distant groups in outlier substitution were observed in any block (*z*s < |1.06|, *p*s ≥ .292). This indicates that the trimming procedure did not differentially affect groups.

**Table S3**

*Z Tests for Differences in Two Proportions on Proportional Deviation Above the Shortest Route and Map Completion Time in Experiment 1*

|  |  | Training Block | |  | Training-Consistent Test Block | |  | Training-Inconsistent Test Block | |
| --- | --- | --- | --- | --- | --- | --- | --- | --- | --- |
| Measure | Group Comparison | *z* | *p* |  | *z* | *p* |  | *z* | *p* |
| Proportional Deviation Above the Shortest Route | pen→no-hand vs. no-hand→pen | 0.06 | .950 |  | - | - |  | 1.05 | .293 |
|  | pen→no-pen vs. no-pen→pen | - | - |  | -0.98 | .328 |  | - | - |
| Map Completion Time | pen→no-pen vs. no-pen→pen | 1.04 | .299 |  | - | - |  | -0.99 | .321 |

In sum, findings indicated that extreme values were rare and evenly distributed across groups, so that the trimming procedure was unlikely to have introduced systematic bias into the analyses reported in the article.

**S3. Experiment 1: Additional Exploratory Analyses on Offloading Strategies**

To assess whether participants trained with pen use allowed (i.e., pen→no-pen and pen→no-hand groups) actually employed offloading strategies during the training, we conducted two one-sample *t*-tests against zero, one for each pen group, on the mean number of strategies used in this stage. Participants in the pen→no-pen group employed on average 3.47 offloading strategies (*SD* = 0.78), a value significantly greater than zero, *t*(34) = 26.22, *p* < .001, *d* = 4.43. Similarly, participants in the pen→no-hand group employed an average of 3.27 strategies (*SD* = 1.10), also significantly above zero, *t*(35) = 19.45, *p* < .001, *d* = 3.24.

Because both groups completed the training under the same experimental condition (pen use allowed), we additionally examined whether they differed in their initial adoption of offloading strategies. An independent-samples *t*-test indicated that the pen→no-pen and pen→no-hand groups did not significantly differ in the number of strategies employed, *t*(69) = 0.85, *p* = .348, *d* = .22. These findings indicate that participants in both groups allowed to use the pen during the training actually relied on offloading strategies in this stage and did so to a comparable extent.

We next focused on the influence of prior training experience on the test stage. Based on previous evidence indicating that prior experience with offloading strategies increases the likelihood of their subsequent use (Scarampi & Gilbert, 2020; Florean et al., 2025), we examined whether being trained on the route planning task with vs. without the opportunity to offload cognition via pen use leads to different degrees of offloading strategy adoption in the test stage. We expected that participants trained with pen use available would use more offloading strategies in the test block allowing pen use than those trained without it.

We conducted a one-way ANOVA on the average number of offloading strategies devised in this test block. Groups significantly differed in their use of offloading strategies, *F*(3,133) = 11.01, *p* < .001, η²_p_ = 0.20. Tukey’s post-hoc comparisons indicated that participants in the pen→no-pen group (*M* = 3.52, *SD* = 0.73) used more strategies than those in the no-pen→pen group (*M* = 2.46, *SD* = 0.87), *t*(133) = 4.58, *p* < .001, *d* = 1.11, and the no-hand→pen group (*M* = 2.56, *SD* = 1.22), *t*(133) = 4.19, *p* < .001, *d* = 1.02. Similarly, participants in the pen→no-hand group (*M* = 3.35, *SD* = 0.94) used more strategies than those in the no-pen→pen group, *t*(133) = 3.86, *p* < .001, *d* = .93, and the no-hand→pen group, *t*(133) = 3.47, *p* = .004, *d* = .84. The other post-hoc comparisons were not statistically significant (*t*s < 0.77, *p*s > .871). Results remained unchanged after controlling for test block order.^[[1]](#footnote-1)^ Figure S1 provides a graphical representation of the number of offloading strategies used as a function of the group.

Four additional one-way ANOVAs, each focused on one of the four categories of offloading strategies, were conducted to determine whether specific strategies were more frequently used by participants trained with the pen compared to those trained without it.

**Figure S1**

*Use of Offloading Strategies in the Test Block Allowing Pen Use as a Function of the Group*

*Note*. Bars represent standard errors.

Groups did not differ in the proportion of test maps with starting and arrival places marked out (pen→no-pen group, *M* = 0.96, *SD* = 0.18, *vs*. pen→no-hand group, *M* = 0.92, *SD* = 0.28, *vs*. no-pen→pen group, *M* = 0.87, *SD* = 0.33, *vs*. no-hand→pen group, *M* = 0.79, *SD* = 0.42), *F*(3,133) = 1.94, *p* = .126, η²_p_ = 0.04, nor in the proportion of maps with intermediate locations marked out (pen→no-pen group, *M* = 0.80, *SD* = 0.39, *vs*. pen→no-hand group, *M* = 0.83, *SD* = 0.38, *vs*. no-pen→pen group, *M* = 0.59, *SD* = 0.48, *vs*. no-hand→pen group, *M* = 0.67, *SD* = 0.45), *F*(3,133) = 2.54, *p* = .059, η²_p_ = 0.05. However, significant between-group differences were observed in the proportion of test maps with ordering rules marked out, *F*(3,133) = 8.92, *p* < .001, η²_p_ = 0.17, and plan progression tracked, *F*(3,133) = 5.90, *p* < .001, η²_p_ = 0.12. Tukey’s post-hoc comparisons indicated that participants in the pen→no-pen group (*M* = 0.88, *SD* = 0.32) marked the ordering rules in a higher proportion of maps than those in the no-pen→pen group (*M* = 0.39, *SD* = 0.44), *t*(133) = 4.75, *p* < .001, *d* = 1.15, and the no-hand→pen group (*M* = 0.54, *SD* = 0.46), *t*(133) = 3.36, *p* = .006, *d* = .82. Participants in the pen→no-hand group (*M* = 0.74, *SD* = 0.44) also marked the rules more frequently than those in the no-pen→pen group, *t*(133) = 3.44, *p* = .004, *d* = .83, though not more frequently than those in the no-hand→pen group, *t*(133) = 2.04, *p* = .179, *d* =.49. Similarly, participants in the pen→no-pen group (*M* = 0.89, *SD* = 0.32) tracked their plan on a higher proportion of test maps than those in the no-pen→pen group (*M* = 0.62, *SD* = 0.45), *t*(133) = 2.80, *p* = .029, *d* = .68, and the no-hand→pen group (*M* = 0.57, *SD* = 0.45), *t*(133) = 3.33, *p* = .006, *d* = .81. Participants in the pen→no-hand group (*M* = 0.86, *SD* = 0.35) also tracked their plan progression more frequently than those in the no-hand→pen group, *t*(133) = 3.09, *p* = .013, *d* = .75, though the difference was only marginally significant compared to those in the no-pen→pen group, *t*(133) = 2.57, *p* = .055, *d* = .62. All other comparisons were not significant: ordering rules marked out (*t*s < 1.38, *p*s > .517) and for plan progress tracked (*t*s < 0.53, *p*s > .954).

Therefore, during the test stage, participants trained with the pen employed more offloading strategies compared to those trained without it. Specifically, participants trained with pen use relied more frequently on two offloading strategies: marking out ordering rules and tracking plan progression. These findings are consistent with the strategy perseveration effect observed in previous studies (Scarampi & Gilbert, 2020; Florean et al., 20252025), and served as a guide for the design of visual aids supporting offloading strategies and Experiment 2.

However, strategy perseveration *per se* cannot explain the asymmetric transfer effect observed in test blocks of Experiment 1. Indeed, we expected that prior experience (vs. no experience) with offloading during the training stage would influence the extent of slowdown (vs. speedup) in the test stage even beyond the degree of reliance on offloading strategies during this stage. We therefore conducted an exploratory ANCOVA on Δ map completion time, with group as a between-subjects factor and the number of offloading strategies used in the test stage as a covariate. Additionally, based on the observed strategy perseveration effect, we included the interaction between group and offloading strategies in the model to account for the possibility that the impact of prior training experience on Δ map completion time might differ depending on the extent to which participants relied on offloading strategies during the test stage.

The effect of offloading strategies used was significant, *F*(1,128) = 4.14, *p* = .044, η²_p_ = .03, and the group × offloading strategies interaction was also significant, *F*(3,128) = 2.78, *p* = .044, η²_p_ = .06. Importantly, group remained a significant predictor, *F*(3,128) = 3.05, *p* = .031, η²_p_ = .07. Between-group post-hoc comparisons revealed that participants in the pen→no-pen group had significantly higher Δ map completion times than those in both the no-pen→pen group, *t*(128) = 2.92, *p* = .004, *d* = .86, and the no-hand→pen group, *t*(128) = 2.05, *p* = .043, *d* = .58. Similarly, participants in the pen→no-hand group had significantly higher Δ map completion times than those in the no-pen→pen group, *t*(128) = 2.19, *p* = .030, *d* = .59, although not higher than those in the no-hand→pen group, *t*(128) = 1.21, *p* = .228, *d* = .31. The remaining between-group comparisons were not statistically significant (*t*s < |1.04|, ps > .304).

Thus, even after controlling for the number of offloading strategies used during the test stage, participants trained with the pen exhibited a greater slowdown when pen use was removed compared to the speedup observed in those trained without the pen when it was introduced, consistent with H3. Specifically, this pattern was consistently observed for participants in the pen→no-pen group and partially observed for participants in the pen→no-hand group. Given the relatively modest sample size for this model, these ANCOVA results should be interpreted cautiously.

**S4. Experiment 1: Route Planning Task Performance in the Training Stage**

In the training stage of Experiment 1, participants completed the route planning task under one of three between-participants conditions (pen, no-pen, and no-hand). We conducted a series of one-way ANOVAs on the performance and perception measures of the route planning task, with the training condition (pen, no-pen, no-hand) as the between-participants factor. The results are reported in Tables S4–S7.

**Table S4**

| Dependent Variable | SS | MS | *F*(2,134) | *p* | η²_p_ |
| --- | --- | --- | --- | --- | --- |
| Proportional deviation above the shortest route | 0.00 | 0.00 | 0.42 | .655 | .01 |
| Map Completion Time | 34814.74 | 17407.37 | 4.35 | .015 | .06 |
| Perceived Task Difficulty | 22.71 | 11.36 | 10.34 | <.001 | .13 |
| Perceived Task Effort | 34.42 | 17.21 | 14.55 | <.001 | .18 |

*One-Way ANOVAs for the Effect of Training Condition on Performance and Perception Measures in the Training Stage of the Route Planning Task*

*Note*. SS = sum of squares; MS = mean Square; η²ₚ = partial eta squared.

**Table S5**

*Tukey’s Post Hoc Comparisons for the Effect of Training Condition on Map Completion Time in the Training Stage*

| Comparison | | | Mean Difference | *SE* | *df* | *t* | *p* | Cohen's *d* |
| --- | --- | --- | --- | --- | --- | --- | --- | --- |
| pen | vs. | no-pen | -39.25 | 13.33 | 134 | -2.94 | 0.011 | -0.62 |
| pen | vs. | no-hand | -10.46 | 13.33 | 134 | -0.78 | 0.713 | -0.17 |
| no-pen | vs. | no-hand | 28.79 | 15.58 | 134 | 1.85 | 0.158 | 0.46 |

*Note.* SE = standard error.

**Table S6**

*Tukey’s Post Hoc Comparisons for the Effect of Training Condition on Perceived Task Difficulty in the Training Stage*

| Comparison | | | Mean Difference | *SE* | *df* | *t* | *p* | Cohen's *d* |
| --- | --- | --- | --- | --- | --- | --- | --- | --- |
| pen | vs. | no-pen | -0.86 | 0.22 | 134 | -3.89 | <.001 | -0.82 |
| pen | vs. | no-hand | -0.77 | 0.22 | 134 | -3.47 | 0.002 | -0.73 |
| no-pen | vs. | no-hand | 0.09 | 0.26 | 134 | 0.35 | 0.934 | 0.09 |

*Note.* SE = standard error.

**Table S7**

*Tukey’s Post Hoc Comparisons for the Effect of Training Condition on Perceived Task Effort in the Training Stage*

| Comparison | | | Mean Difference | *SE* | *df* | *t* | *p* | Cohen's *d* |
| --- | --- | --- | --- | --- | --- | --- | --- | --- |
| pen | vs. | no-pen | -1.06 | 0.23 | 134 | -4.63 | <.001 | -0.97 |
| pen | vs. | no-hand | -0.94 | 0.23 | 134 | -4.10 | <.001 | -0.86 |
| no-pen | vs. | no-hand | 0.12 | 0.27 | 134 | 0.45 | 0.893 | 0.11 |

*Note.* SE = standard error.

To summarize, in the training stage, no significant differences were observed in route planning accuracy (i.e., proportional deviation above the shortest route) between training conditions. However, conditions differed in the time required to complete a map: participants with the opportunity to offload cognition via pen use completed routes faster than those in the no-pen condition. Pen use also resulted in lower perceived task difficulty and effort compared to both the no-pen and no-hand conditions.

**S5. Experiment 1: Transfer Effects on Route Planning Task Performance Controlling for Test Block Order**

To assess whether findings on transfer effects remained unchanged after controlling for test block order, we conducted two 4 (Group: pen→no-pen vs. pen→no-hand vs. no-pen→pen vs. no-hand→pen) × 2 (Test block: training-consistent vs. training-inconsistent) × 2 (Test block order: training-consistent first vs. training-inconsistent first) mixed ANOVAs, one for each performance measure of the maps completed without errors. For the same reason, we also repeated the four key planned comparisons across test blocks, as well as the ANOVA on Δ map completion times, controlling for test block presentation order. Results are reported in Tables S8–S10.

**Table S8**

*Mixed ANOVAs on Route Planning Task Performance Measures Controlling for Test Block Order*

| Dependent variable | Factor | SS | MS | *df* | *F* | *p* | η²_p_ |
| --- | --- | --- | --- | --- | --- | --- | --- |
| Proportional Deviation Above the Shortest Route | Group | 0.02 | 0.01 | 3,128 | 0.27 | .846 | .01 |
|  | Test Block | 0.01 | 0.01 | 1,128 | 1.78 | .184 | .01 |
|  | Test Block Order | 0.02 | 0.02 | 1,128 | 1.15 | .286 | .01 |
|  | Group × Test Block | 0.17 | 0.06 | 3,128 | 8.13 | <.001 | .16 |
|  | Group × Test Block Order | 0.02 | 0.01 | 3,128 | 0.41 | .743 | .01 |
|  | Test Block × Test Block Order | 0.00 | 0.00 | 1,128 | 0.03 | .871 | .00 |
|  | Group × Test Block × Test Block Order | 0.01 | 0.00 | 3,128 | 0.68 | .566 | .02 |
| Map Completion Time | Group | 4841.07 | 1613.69 | 3,128 | 0.26 | .851 | .01 |
|  | Test Block | 28102.21 | 28102.21 | 1,128 | 16.48 | <.001 | .11 |
|  | Test Block Order | 3094.30 | 3094.30 | 1,128 | 0.51 | .478 | .00 |
|  | Group × Test Block | 173909.25 | 57969.75 | 3,128 | 34.00 | <.001 | .44 |
|  | Group × Test Block Order | 8339.91 | 2779.97 | 3,128 | 0.45 | .715 | .01 |
|  | Test Block × Test Block Order | 556.86 | 556.86 | 1,128 | 0.33 | .569 | .00 |
|  | Group × Test Block × Test Block Order | 821.11 | 273.7 | 3,128 | 0.16 | .923 | .00 |

*Note.* SS = sum of squares; MS = mean square; η²ₚ = partial eta squared.

**Table S9**

*Planned Comparisons for Groups Across Test Blocks Controlling for Test Block Order*

| Dependent Variable | Comparison | Group | Mean  Difference | *SE* | *df* | *t* | *p* | Cohen's *d* |
| --- | --- | --- | --- | --- | --- | --- | --- | --- |
| Proportional Deviation Above the Shortest Route | Training-Inconsistent Test Block vs. Training-Consistent Test Block | pen→no-pen | 0.06 | 0.02 | 128 | 2.89 | .004 | .50 |
|  |  | pen→no-hand | 0.07 | 0.02 | 128 | 3.41 | <.001 | .57 |
|  |  | no-pen→pen | -0.03 | 0.02 | 128 | -1.21 | .230 | -.21 |
|  |  | no-hand→pen | -0.05 | 0.02 | 128 | -2.27 | .025 | -.40 |
| Map Completion Time | Training-Inconsistent Test Block vs. Training-Consistent Test Block | pen→no-pen | 74.99 | 10.03 | 128 | 7.47 | <.001 | 1.28 |
|  |  | pen→no-hand | 66.93 | 9.73 | 128 | 6.88 | <.001 | 1.15 |
|  |  | no-pen→pen | -30.25 | 10.17 | 128 | -2.97 | .004 | -.52 |
|  |  | no-hand→pen | -30.25 | 10.17 | 128 | -2.97 | .004 | -.52 |

*Note.* SE = standard error.

**Table S10**

*ANOVA and Between-Group Comparisons on Δ Map Completion Time Controlling for Test Block Order*

| Factor | | | SS | | MS | *df* | *F* | *p* | η²_p_ |
| --- | --- | --- | --- | --- | --- | --- | --- | --- | --- |
| Group | | | 57025.45 | | 19008.48 | 3,128 | 5.57 | .001 | 0.12 |
| Test Block Order | | | 492.3 | | 492.3 | 1,128 | 0.14 | .705 | 0.00 |
| Group × Test Block Order | | | 2252.86 | | 750.95 | 3,128 | 0.22 | .882 | 0.01 |
| Comparison |  |  | | Mean Difference | *SE* | *df* | *t* | *p* | Cohen's *d* |
| pen→no-pen | vs. | pen→no-hand | | -8.06 | 13.98 | 128 | -0.58 | .565 | .14 |
| pen→no-pen | vs. | no-pen→pen | | -44.74 | 14.29 | 128 | -3.13 | .002 | .77 |
| pen→no-pen | vs. | no-hand→pen | | -44.73 | 14.29 | 128 | -3.13 | .002 | .77 |
| pen→no-hand | vs. | no-pen→pen | | -36.69 | 14.08 | 128 | -2.61 | .010 | .63 |
| pen→no-hand | vs. | no-hand→pen | | -36.68 | 14.08 | 128 | -2.61 | .010 | .63 |
| no-pen→pen | vs. | no-hand→pen | | 0.01 | 14.38 | 128 | 0.00 | 1.000 | .00 |

*Note.* SS = sum of squares; MS = mean square; η²ₚ = partial eta squared; SE = standard error.

The group × test block interaction and all four planned comparisons across test blocks for both proportional deviation and map completion time remained unchanged after controlling for test block order, as well as the ANOVA and the between-group comparisons on Δ map completion time.

**S6. Experiment 1: Moderation Analysis on Δ Map Completion Time Using the Pen→No-Hand Group as Reference**

To check the robustness of the moderation analysis reported in the article for Experiment 1, we re-ran the General Linear Model (GLM) on Δ map completion times between test blocks allowing and precluding pen use, this time setting the pen→no-hand group as the reference level instead of the pen→no-pen group.

The simple effects analyses showed that, like participants in the pen→no-pen group, those in the pen→no-hand group had significantly greater Δ map completion times than those in the no-pen→pen group, *t*(127) = 2.85, *p* = .005, *d* = .69, and the no-hand→pen group, *t*(127) = 3.68, *p* < .001, *d* = .89, when spatial working memory scores were 1 *SD* below the mean. At the spatial working memory mean, the pen→no-hand group again showed higher Δ map completion times than both the no-pen→pen group, *t*(127) = 2.94, *p* = .004, *d* = .71, and the no-hand→pen group, *t*(127) = 3.09, *p* = .002, *d* =.75. At 1 *SD* above the mean, no significant between-group differences were observed (*t*s < |1.39|, *p*s > .170).

These findings are consistent with those obtained when using the pen→no-pen group as the reference and support H4, showing that higher spatial working memory buffers the cost of the removal of offloading opportunities in both groups trained with pen use allowed.

**S7. Experiment 1: Transfer Effects on Perceived Task Difficulty and Effort**

To test the hypothesis that the removal of pen use would lead to an increase in perceived task difficulty and effort (H5), while its introduction would result in a decrease in these perceptions (H6), we carried out 4 (pen→no-pen *vs*. pen→no-hand *vs*. no-pen→pen *vs*. no-hand→pen groups) x 2 (training-consistent *vs*. training-inconsistent test blocks) mixed ANOVAs on perceived task difficulty and effort, followed by the four within-group key comparisons across test blocks. A significant interaction between group and test block was observed for the perceived difficulty of the route planning task, *F*(3, 133) = 58.18, *p* < .001, η²ₚ = .57. The main effect of group was also statistically significant, *F*(3, 133) = 3.02, *p* = .032, η²ₚ = .06, while the main effect of test block was not, *F*(1, 133) = 2.45, *p* = .120, η²ₚ = .02. Participants in both the pen→no-pen group and the pen→no-hand group rated the task as more difficult in the training-inconsistent block than in the training-consistent block, *t*(133) = 8.01, *p* < .001, *d* = 1.27, and *t*(133) = 6.67, *p* < .001, *d* = .95, respectively. Conversely, participants in the no-pen→pen group and the no-hand→pen group perceived the task as less difficult in the training-inconsistent block compared to the training-consistent block, *t*(133) = -3.48, *p* < .001, *d* = -.74, and *t*(133) = -7.61, *p* < .001, *d* = -1.50, respectively.

A similar pattern of results was found for task perceived effort. Specifically, the interaction between group and test block was statistically significant, *F*(3, 133) = 35.92, *p* < .001, η²ₚ = .45. The main effect of group was also statistically significant, *F*(3, 133) = 3.89, *p* = .011, η²ₚ = .08, whereas the main effect of test block was not, *F*(1, 133) = 0.05, *p* = .819, η²ₚ = .00. Participants in the pen→no-pen group and the pen→no-hand group reported significantly more effort to complete the training-inconsistent block compared to the training-consistent block, *t*(133) = 5.33, *p* < .001, *d* = .84, and *t*(133) = 5.36, *p* < .001, *d* = .75, respectively. Conversely, participants in the no-pen→pen group and the no-hand→pen group reported significantly less effort in the training-inconsistent block compared to the training-consistent block, *t*(133) = -3.81, *p* < .001, *d* = -.77, and *t*(133) = -6.05, *p* < .001, *d* = 1.29, respectively.

To assess whether findings on transfer effects on the perception of the planning task in Experiment 1 remained unchanged after controlling for test block order, we conducted two 4 (Group: pen→no-pen vs. pen→no-hand vs. no-pen→pen vs. no-hand→pen) × 2 (Test block: training-consistent vs. training-inconsistent) × 2 (Test block order: training-consistent first vs. training-inconsistent first) mixed ANOVAs, one on perceived task difficulty, and one on perceived task effort. The four within-group planned comparisons across test blocks were also repeated controlling for test block order. Results are reported in Tables S11–S12.

**Table S11**

*Mixed ANOVAs on Perceived Task Difficulty and Effort Controlling for Test Block Order*

| Dependent variable | Factor | SS | MS | *df* | *F* | *p* | η²_p_ |
| --- | --- | --- | --- | --- | --- | --- | --- |
| Perceived Task Difficulty | Group | 16.5 | 5.5 | 3,129 | 3.02 | .032 | .07 |
|  | Test Block | 2.25 | 2.25 | 1,129 | 2.41 | .123 | .02 |
|  | Test Block Order | 4.75 | 4.75 | 1,129 | 2.61 | .109 | .02 |
|  | Group × Test Block | 158.44 | 52.81 | 3,129 | 56.59 | <.001 | .57 |
|  | Group × Test Block Order | 1.81 | 0.6 | 3,129 | 0.33 | .803 | .01 |
|  | Test Block × Test Block Order | 0.06 | 0.06 | 1,129 | 0.06 | .806 | .00 |
|  | Group × Test Block × Test Block Order | 0.77 | 0.26 | 3,129 | 0.28 | .843 | .01 |
| Perceived Task Effort | Group | 23.27 | 7.76 | 3,129 | 3.85 | .011 | .08 |
|  | Test Block | 0.07 | 0.07 | 1,129 | 0.06 | .809 | .00 |
|  | Test Block Order | 4.19 | 4.19 | 1,129 | 2.08 | .152 | .02 |
|  | Group × Test Block | 129.9 | 43.3 | 3,129 | 35.1 | <.001 | .45 |
|  | Group × Test Block Order | 1.59 | 0.53 | 3,129 | 0.26 | .852 | .01 |
|  | Test Block × Test Block Order | 0.37 | 0.37 | 1,129 | 0.3 | .586 | .00 |
|  | Group × Test Block × Test Block Order | 1.22 | 0.41 | 3,129 | 0.33 | .804 | .01 |

*Note.* SS = sum of squares; MS = mean square; η²ₚ = partial eta squared.

**Table S12**

*Planned Comparisons for Groups Across Test Blocks on Task Perceived Difficulty and Effort Controlling for Test Block Order*

| Dependent Variable | Comparison | Group | Mean Difference | *SE* | *df* | *t* | *p* | Cohen's *d* |
| --- | --- | --- | --- | --- | --- | --- | --- | --- |
| Task Perceived Difficulty | Training-Inconsistent Test Block vs. Training-Consistent Test Block | pen→no-pen | -1.83 | 0.23 | 129 | 7.88 | <.001 | 1.33 |
|  |  | pen→no-hand | -1.5 | 0.23 | 129 | 6.59 | <.001 | 1.10 |
|  |  | no-pen→pen | 0.81 | 0.24 | 129 | -3.41 | <.001 | -.59 |
|  |  | no-hand→pen | 1.79 | 0.24 | 129 | -7.52 | <.001 | -1.31 |
| Task Perceived Effort | Training-Inconsistent Test Block vs. Training-Consistent Test Block | pen→no-pen | -1.4 | 0.27 | 129 | 5.26 | <.001 | .89 |
|  |  | pen→no-hand | -1.39 | 0.26 | 129 | 5.31 | <.001 | .89 |
|  |  | no-pen→pen | 1.03 | 0.27 | 129 | -3.76 | <.001 | -.65 |
|  |  | no-hand→pen | 1.63 | 0.27 | 129 | -5.97 | <.001 | -1.04 |

*Note.* SE = standard error.

The group × test block interaction and the planned within-group comparisons on both perceived task difficulty and perceived task effort remained unchanged after controlling for test block order.

**S8. Experiment 2: Outlier Detection and Exploratory Analysis on Group Differences in Outlier Substitution**

As part of the data preprocessing for Experiment 2, we implemented an outlier identification and trimming procedure analogous to that adopted in Experiment 1. For each performance measure from the route planning task analysed in Experiment 2, observations were classified as outliers if they fell outside ±3 *SD* from the mean. This criterion was applied separately for each experimental group and block (Del Missier et al., 2021; Florean et al., 2025; Miyake et al., 2000). Specifically, this procedure was applied to proportional deviation above the shortest route and map completion time, as in Experiment 1, as well as to solution-typing time. Tables S13–S15 report means and standard deviations for the raw (untrimmed) data, lower and upper cutoffs (*M* ± 3 *SD*) used for outlier identification, and the percentages of outlier substitution for each group and block.

**Table S13**

*Descriptive Statistics for Raw Data and Percentages of Outlier Substitution for Proportional Deviation Above the Shortest Route in Experiment 2*

|  | **Training Block** | | | | **Test Block** | | | |
| --- | --- | --- | --- | --- | --- | --- | --- | --- |
|  |  | Thresholds | |  |  | Thresholds | |  |
| **Group** | *M* (*SD*) | Lower (*M* - *3 SD*) | Upper (*M* + 3 *SD*) | % Outlier Substitution | *M* (*SD*) | Lower (*M* - 3 *SD*) | Upper (*M* + 3 *SD*) | % Outlier Substitution |
| **Full Offloading ➝ No Offloading** | 0.10 (0.06) | -0.08 | 0.28 | 2.94% | 0.18 (0.15) | -0.27 | 0.63 | 0.00% |
| **Partial Offloading ➝ No Offloading** | 0.10 (0.05) | -0.05 | 0.25 | 3.03% | 0.18 (0.14) | -0.24 | 0.60 | 3.03% |
| **No Offloading ➝ Full Offloading** | 0.11 (0.05) | -0.04 | 0.26 | 5.88% | 0.09 (0.06) | -0.09 | 0.27 | 3.03% |
| **No Offloading ➝ Partial Offloading** | 0.13 (0.07) | -0.08 | 0.34 | 2.94% | 0.11 (0.05) | -0.04 | 0.26 | 0.00% |
| **No Offloading ➝ No Offloading** | 0.11 (0.07) | -0.10 | 0.32 | 0.00% | 0.14 (0.10) | -0.16 | 0.44 | 3.23% |

**Table S14**

*Descriptive Statistics for Raw Data and Percentages of Outlier Substitution for Map Completion Time in Experiment 2*

|  | **Training Block** | | | | **Test Block** | | | |
| --- | --- | --- | --- | --- | --- | --- | --- | --- |
|  |  | Thresholds | |  |  | Thresholds | |  |
| **Group** | *M* (*SD*) | Lower (*M* - 3 *SD*) | Upper (*M* + 3 *SD*) | % Outlier Substitution | *M* (*SD*) | Lower (*M* - 3 *SD*) | Upper (*M* + 3 *SD*) | % Outlier Substitution |
| **Full Offloading ➝ No Offloading** | 145.99 (59.86) | -33.59 | 325.57 | 0.00% | 268.01 (114.15) | -74.44 | 610.46 | 3.13% |
| **Partial Offloading ➝ No Offloading** | 140.57 (64.81) | -53.86 | 335.00 | 0.00% | 253.11 (102.84) | -55.41 | 561.63 | 0.00% |
| **No Offloading ➝ Full Offloading** | 204.03 (80.10) | -36.27 | 444.33 | 0.00% | 143.36 (59.66) | -35.62 | 322.34 | 3.03% |
| **No Offloading ➝ Partial Offloading** | 209.58 (70.61) | -2.25 | 421.41 | 2.94% | 159.04 (64.36) | -34.04 | 352.12 | 0.00% |
| **No Offloading ➝ No Offloading** | 236.32 (85.87) | -21.29 | 493.93 | 0.00% | 217.98 (81.71) | -27.15 | 463.11 | 0.00% |

*Note.* Mean (*M*), Standard Deviation (*SD*), and Threshold values are expressed in seconds.

**Table S15**

*Descriptive Statistics for Raw Data and Percentages of Outlier Substitution for Solution-Typing Time in Experiment 2*

|  | **Training Block** | | | | **Test Block** | | | |
| --- | --- | --- | --- | --- | --- | --- | --- | --- |
|  |  | Thresholds | |  |  | Thresholds | |  |
| **Group** | *M* (*SD*) | Lower (*M* - 3 *SD*) | Upper (*M* + 3 *SD*) | % Outlier Substitution | *M* (*SD*) | Lower (*M* - 3 *SD*) | Upper (*M* + 3 *SD*) | % Outlier Substitution |
| **Full Offloading ➝ No Offloading** | 44.06 (26.98) | -36.88 | 125.00 | 0.00% | 90.64 (59.40) | -87.56 | 268.84 | 3.13% |
| **Partial Offloading ➝ No Offloading** | 57.06 (37.10) | -54.24 | 168.36 | 3.03% | 86.09 (71.57) | -128.62 | 300.80 | 3.03% |
| **No Offloading ➝ Full Offloading** | 54.92 (46.89) | -85.75 | 195.59 | 0.00% | 35.71 (21.08) | -27.53 | 98.95 | 3.03% |
| **No Offloading ➝ Partial Offloading** | 54.27 (39.14) | -63.15 | 171.69 | 2.94% | 39.51 (21.36) | -24.57 | 103.59 | 2.94% |
| **No Offloading ➝ No Offloading** | 64.20 (50.04) | -85.92 | 214.32 | 3.23% | 59.58 (42.13) | -66.81 | 185.97 | 3.23% |

*Note.* Mean (*M*), Standard Deviation (*SD*), and Threshold values are expressed in seconds.

Overall, the proportion of values identified as outliers was low across all measures, groups, and blocks, indicating that extreme values were relatively rare in the dataset. Therefore, for each performance measure and block, we compared the two groups exhibiting the highest and lowest proportions of outlier substitution using *z*-tests for differences between two proportions, following the same approach adopted in Experiment 1. Test statistics are reported in Table S16.

**Table S16**

| **Measure** | **Group Comparison** | **Training Block** | |  | **Test Block** | | |
| --- | --- | --- | --- | --- | --- | --- | --- |
|  |  | *z* | *p* |  | *z* | *p* |  |
| **Proportional Deviation Above the Shortest Route** | Full Offloading ➝ No Offloading vs. No Offloading ➝ No Offloading | - | - |  | 1.02 | .306 |  |
|  | No Offloading ➝ Full Offloading vs. No Offloading ➝ No Offloading | -1.37 | .170 |  | - | - |  |
| **Map Completion Time** | Full Offloading ➝ No Offloading vs. No Offloading ➝ No Offloading | - | - |  | -0.99 | .321 |  |
|  | No Offloading ➝ Partial Offloading vs. No Offloading ➝ No Offloading | -0.96 | .336 |  | - | - |  |
| **Solution-Typing Time** | Full Offloading ➝ No Offloading vs. No Offloading ➝ No Offloading | 1.06 | .291 |  | - | - |  |
|  | No Offloading ➝ Partial Offloading vs. No Offloading ➝ No Offloading | - | - |  | 0.07 | .947 |  |

Across all measures and blocks, none of these comparisons reached statistical significance (*z*s < |1.38|, *p*s > .169), indicating that the frequency of outlier substitution did not differ significantly between experimental groups. To summarize, the results indicate that the trimming procedure in Experiment 2 affected only a small proportion of observations and was unlikely to have introduced systematic biases into the analyses reported in the article.

**S9. Experiment 2: Transfer Effects on Route Planning Task Performance Controlling for Verbal Working Memory**

To assess whether the findings on transfer effects in Experiment 2 remained unchanged after controlling for verbal working memory, we repeated the series of 5 (Group: full offloading → no offloading vs. partial offloading → no offloading vs. no offloading → full offloading vs. no offloading → partial offloading vs. no offloading → no offloading) × 2 (Block: training vs. test) mixed ANOVAs on route planning task performance measures, entering Backward Digit Span scores as a covariate. All planned comparisons were also repeated controlling for verbal working memory. Finally, an ANCOVA on Δ map completion time was conducted with group as a factor and Backward Digit Span as a covariate. Results are reported in Tables S17–S19.

**Table S17**

*Mixed ANOVAs on Route Planning Task Performance with Verbal Working Memory as a Covariate*

| Dependent variable | Factor | SS | MS | *df* | *F* | *p* | η²_p_ |
| --- | --- | --- | --- | --- | --- | --- | --- |
| Proportional Deviation Above the Shortest Route | Group | 0.07 | 0.02 | 4,156 | 1.66 | .161 | .04 |
|  | Block | 0.07 | 0.07 | 1,156 | 13.93 | <.001 | .08 |
|  | verbal WM | 0.03 | 0.03 | 1,156 | 3.25 | .073 | .02 |
|  | Group × Block | 0.21 | 0.05 | 4,156 | 10.99 | <.001 | .22 |
|  | Block × verbal WM | 0.04 | 0.04 | 1,156 | 8.36 | .004 | .05 |
| Map Completion Time | Group | 123747.93 | 30937 | 4,156 | 3.42 | .010 | .08 |
|  | Block | 5638.16 | 5638.16 | 1,156 | 1.66 | .199 | .01 |
|  | verbal WM | 5052.98 | 5052.98 | 1,156 | 0.56 | .456 | .00 |
|  | Group × Block | 501801.24 | 125450 | 4,156 | 37.05 | <.001 | .49 |
|  | Block × verbal WM | 829.51 | 829.51 | 1,156 | 0.24 | .621 | .00 |

*Note.* SS = sum of squares; MS = mean square; Verbal WM = verbal working memory, measured with Backward Digit Span; η²ₚ = partial eta squared.

**Table S18**

*Planned Comparisons on Route Planning Task Performance Measures with Verbal Working Memory as a Covariate*

| Dependent Variable | Comparison | | | | MD | *SE* | *df* | *t* | *p* | Cohen's *d* |
| --- | --- | --- | --- | --- | --- | --- | --- | --- | --- | --- |
|  | Group | | Block | |  |  |  |  |  |  |
| Proportional Deviation Above the Shortest Route | full offloading → no offloading |  | Training | vs. Test | -0.08 | 0.02 | 156 | -4.35 | <.001 | -.77 |
|  | partial offloading → no offloading |  | Training | vs. Test | -0.09 | 0.02 | 156 | -5.40 | <.001 | -.94 |
|  | no offloading → full offloading |  | Training | vs. Test | 0.03 | 0.02 | 156 | 1.64 | .102 | .29 |
|  | no offloading → partial offloading |  | Training | vs. Test | 0.03 | 0.02 | 156 | 1.56 | .121 | .27 |
|  | no offloading → no offloading |  | Training | vs. Test | -0.02 | 0.02 | 156 | -1.38 | .170 | -.25 |
|  | full offloading → no offloading | vs. partial offloading → no offloading | Training |  | 0.00 | 0.02 | 156 | -0.25 | .804 | -.06 |
|  | no offloading → full offloading | vs. no offloading → partial offloading | Test |  | -0.02 | 0.03 | 156 | -0.72 | .471 | -.18 |
|  | full offloading → no offloading | vs. no offloading → no offloading | Training |  | 0.02 | 0.01 | 156 | 1.46 | .147 | .32 |
|  | partial offloading → no offloading |  | Test |  | -0.04 | 0.02 | 156 | -1.79 | .075 | -.40 |
| Map Completion Time | full offloading → no offloading |  | Training | vs. Test | -116.10 | 14.75 | 156 | -7.87 | <.001 | -1.39 |
|  | partial offloading → no offloading |  | Training | vs. Test | -114.27 | 14.75 | 156 | -7.75 | <.001 | -1.35 |
|  | no offloading → full offloading |  | Training | vs. Test | 59.80 | 14.33 | 156 | 4.17 | <.001 | .73 |
|  | no offloading → partial offloading |  | Training | vs. Test | 50.11 | 14.13 | 156 | 3.55 | <.001 | .61 |
|  | no offloading → no offloading |  | Training | vs. Test | 17.34 | 15.03 | 156 | 1.15 | .251 | .21 |
|  | full offloading → no offloading | vs. partial offloading → no offloading | Training |  | 6.54 | 18.69 | 156 | 0.35 | .727 | .09 |
|  | no offloading → full offloading | vs. no offloading → partial offloading | Test |  | -16.65 | 20.74 | 156 | -0.80 | .423 | -.20 |
|  | full offloading → no offloading | vs. no offloading → no offloading | Training |  | 93.00 | 16.00 | 156 | 5.81 | <.001 | 1.28 |
|  | partial offloading → no offloading |  | Test |  | -39.60 | 18.70 | 156 | -2.11 | .036 | -.47 |

*Note.* MD = Mean Difference, SE = standard error.

**Table S19**

*ANCOVA and Between-Group Comparisons on Δ Map Completion Time with Verbal Working Memory as a Covariate*

| Factor | | | SS | | MS | *df* | *F* | *p* | | η²_p_ |
| --- | --- | --- | --- | --- | --- | --- | --- | --- | --- | --- |
| Group | | | 123883.06 | | 41294.40 | 3,127 | 6.00 | <.001 | | .12 |
| Verbal Working Memory (Backward Digit span) | | | 4049.20 | | 4049.20 | 1,127 | 0.59 | .445 | | .00 |
| Comparison |  | |  | Mean Difference | *SE* | *df* | *t* | *p* | Cohen's *d* | |
| full offloading → no offloading | vs. | partial offloading → no offloading | -0.32 | | 21.63 | 127 | -0.01 | .988 | .00 | |
| full offloading → no offloading | vs. | no offloading → full offloading | 55.30 | | 20.78 | 127 | 2.66 | .009 | .67 | |
| full offloading → no offloading | vs. | no offloading → partial offloading | 66.14 | | 20.52 | 127 | 3.22 | .002 | .80 | |
| partial offloading → no offloading | vs. | no offloading → full offloading | 55.62 | | 20.79 | 127 | 2.68 | .008 | .67 | |
| partial offloading → no offloading | vs. | no offloading → partial offloading | 66.45 | | 20.83 | 127 | 3.19 | .002 | .80 | |
| no offloading → full offloading | vs. | no offloading → partial offloading | 10.83 | | 20.30 | 127 | 0.53 | .595 | .13 | |

*Note.* SE = standard error. ANCOVA was conducted without the no offloading → no offloading group, consistent with the rationale described in the article.

The group × block interaction and all planned comparisons remained unchanged after controlling for verbal working memory on both proportional deviation and map completion time. Likewise, the ANCOVA results on Δ map completion time and the between-group comparisons were unaffected by including verbal working memory as a covariate.

**S10. Experiment 2: Moderation Analysis on Δ Solution-Typing Time Using the Partial Offloading → No Offloading Group as Reference**

In the moderation analysis performed with General Linear Models (GLM) on Δ solution-typing time between the training and test blocks in Experiment 2, we changed the reference level from the full offloading → no offloading group to the partial offloading → no offloading group. This allowed us to test whether both groups trained with external visual aids for offloading strategies showed a less pronounced slowdown in solution-typing time after the removal of these aids when participants had higher verbal working memory capacity.

The simple effects analyses showed that, like participants in the full offloading → no offloading group, those in the partial offloading → no offloading group had significantly greater Δ solution-typing times than those in the no offloading → full offloading group, *t*(124) = 2.82, *p* = .006, *d* = .69, and the no offloading → partial offloading group, *t*(124) = 2.61, *p* = .010, *d* = .64, when verbal working memory scores were 1 SD below the mean. At the mean of verbal working memory scores, participants in the partial offloading → no offloading group showed significantly greater Δ solution-typing times than those in the no offloading → partial offloading group, *t*(124) = 2.21, *p* = .029, *d* = .54, but not compared to those in the no offloading → full offloading group, t(124) = 1.67, *p* = .097, *d* = .41. Finally, when verbal working memory scores were 1 SD above the mean, no significant between-group differences in Δ solution-typing times were observed (*t*s < |0.75|, *p*s > .460). These findings support H4, showing that higher verbal working memory buffers the cost of losing the offloading opportunity on solution-typing time for both the full offloading → no offloading group and the partial offloading → no offloading group.

**S11. Experiment 2: Transfer Effects on Perceived Task Difficulty and Effort**

To investigate whether the removal of visual aids for offloading strategies led to an increase in perceived task difficulty and effort and their introduction led to opposite effects, we ran on these variables the same analyses as for transfer effects on performance. The ANOVA showed a significant main effect of group on perceived task difficulty, *F*(4,161) = 8.35, *p* < .001, η²ₚ = .17, but no significant main effect of block, *F*(1,161) = 0.21, *p* = .646, η²ₚ = .00. However, the group × block interaction was significant, *F*(4,161) = 44.02, *p* < .001, η²ₚ = .52. Participants in the full offloading → no offloading group perceived the task as more difficult in the test block than in the training block, *t*(161) = 7.25, *p* < .001, *d* = 1.03. Similarly, participants in the partial offloading → no offloading group perceived the task as more difficult in the test block than in the training block, *t*(161) = 5.67, *p* < .001, *d* = 1.21. Conversely, participants in the no offloading → full offloading group rated the task as less difficult in the test block than in the training block, *t*(161) = -7.55, *p* < .001, *d* = -1.27. Likewise, participants in the no offloading → partial offloading group perceived the task as easier in the test block than in the training block, *t*(161) = -5.74, *p* < .001, *d* = -.91. Participants in the no offloading → no offloading group did not rate the difficulty of the training block as significantly different from that of the test block, *t*(161) = 1.27, *p* = .208, *d* = .29.

No significant differences were detected in perceived difficulty between the full offloading → no offloading group and the partial offloading → no offloading group in the training block, *t*(161) = 0.00, *p* = 1.000, *d* = .00. However, significant differences were found between the no offloading → full offloading and no offloading → partial offloading groups in the test block, *t*(161) = 2.81, *p* = .006, *d* = .73.

Planned comparisons revealed that participants in the full offloading → no offloading and partial offloading → no offloading groups rated the task as significantly more difficult than participants in the no offloading → no offloading group in the test block, *t*(161) = 2.12, *p* = .035, *d* = .44.

The main effects of group, *F*(4,161) = 9.31, *p* < .001, η²ₚ = .19, and block, *F*(1,161) = 7.90, *p* = .006, η²ₚ = .05, on task perceived effort were qualified by a significant group by block interaction, *F*(4,161) = 50.81, *p* < .001, η²ₚ = .56. Participants in the full offloading → no offloading group perceived they had spent more effort to complete the test block than the training block, *t*(161) = 7.10, *p* < .001, *d* = 1.17. The same was found for participants in the partial offloading → no offloading group, who perceived the task as more effortful in the test block than in the training block, *t*(161) = 4.18, *p* < .001, *d* = .71. In contrast, participants in the no offloading → full offloading group reported less effort to complete the test block compared to the training block, *t*(161) = -9.80, *p* < .001, *d* = -1.60. Similarly, participants in the no offloading → partial offloading group perceived the task as less effortful in the test block than in the training block, *t*(161) = -6.82, *p* < .001, *d* = -1.12. Participants in the no offloading → no offloading group did not rate the perceived effort for the training block as significantly different from that of the test block, *t*(161) = -1.04, *p* = .229, *d* = -.24.

No significant difference in perceived effort was detected between the full offloading → no offloading and partial offloading → no offloading groups in the training block, *t*(161) = 0.34, *p* = .733, *d* = .09. However, participants in the no offloading → full offloading group reported less effort to complete the test block than participants in the no offloading → partial offloading group, *t*(161) = -3.40, *p* < .001, *d* = .77. Participants in the full offloading → no offloading and partial offloading → no offloading groups rated the test block as significantly more effortful than participants in the no offloading → no offloading group, *t*(161) = 2.84, *p* =.005, *d* = .65.

Therefore, in agreement with H5, the removal of visual aids for offloading strategies led to a significant increase in perceived task difficulty and effort for participants trained with these aids. Conversely, in agreement with H6, the introduction of visual aids for offloading strategies decreased these perceptions for participants trained without aids. Additionally, participants in the full offloading → no offloading and partial offloading → no offloading groups rated the test block as more difficult and effortful than those in the no offloading → no offloading group, with these findings highlighting the impact of prior experience with visual aids for offloading on perceived difficulty and effort once these aids were removed. Finally, while participants trained with full or partial aids for offloading strategies did not differ in their perception of task difficulty and effort during the training block, participants provided with aids in the test block reported greater relief in both perceived task difficulty and effort when provided with full aids compared to those provided with partial aids.

**S12. Experiment 2: Assessment of Participants’ Compliance and Vigilance Throughout the Online Experimental Session**

Given that Experiment 2 was conducted online, we assessed participants’ attentional engagement and task compliance throughout the experimental session. To this aim, we conducted an additional 5 (Group: full offloading → no offloading vs. partial offloading → no offloading vs. no offloading → full offloading vs. no offloading → partial offloading vs. no offloading → no offloading) × 2 (Block: training vs. test) mixed ANOVA on the proportion of maps completed without errors. Errors were defined as failures to comply with the planning rules and constraints specified for each map. This measure provides a conservative behavioral index of compliance and sustained attention, as incorrect map completion directly reflects inattention to task instructions.

Critically, we focused on the control group, in which participants completed both the training and the test blocks under identical conditions (i.e., without the opportunity to offload cognition). This group offers the most stringent test of attentional stability, because any change in performance from the training block to the test block cannot be attributed to changes in offloading opportunities, task structure, or strategy availability.

The main effects of Block, *F*(1, 162) = 9.63, *p* = .002, η²ₚ = .06, and Group, *F*(4, 162) = 2.70, *p* = .033, η²ₚ = .06, were qualified by a Block × Group interaction, *F*(4, 162) = 3.64, *p* = .007, η²ₚ = .08. Crucially for the present purpose, participants in the control group did not exhibit a significant decrease in the proportion of maps completed without errors from the training block to the test block, *t*(162) = 1.68, *p* = .095, *d* = .26. This indicates stable task compliance and sustained attention across the duration of the online session for the group in which no change in offloading opportunity occurred.

These findings align with the results reported in the article, showing that the control group did not exhibit significant worsening from training to test in the two primary performance measures of the route planning task: proportional deviation above the shortest route (Section 3.2.2.1) and map completion time (Section 3.2.2.2). The absence of declines in both task compliance and performance-related measures argues against fatigue-related or attentional declines over the course of the experiment.

Finally, the overall pattern of results observed in Experiment 2 closely mirrored that obtained in Experiment 1, which was conducted in the laboratory under direct supervision. This convergence between online and in-lab findings provides further evidence that participants were compliant and attentively engaged throughout the online experimental session.

1. Main effect of group, *F*(3,129) = 10.54, *p* < .001, η²p = 0.20. Post-hoc comparisons: pen→no-pen group *vs*. no-pen→pen group, *t*(129) = 4.46, *p* < .001, *d* = 1.08; pen→no-pen group *vs*. no-hand→pen group *t*(129) = 4.07, *p* < .001; *d* = .99; pen→no-hand group *vs*. no-pen→pen group , *t*(129) = 3.81, *p* = .001, *d* = .92; pen→no-hand group *vs*. no-hand→pen group, *t*(129) = 3.42, *p* = .005, *d* = .82. None of the other effects reached statistical significance (*F*s < 0.31, *p*s > .583). [↑](#footnote-ref-1)
